# Mechanisms of Paclitaxel Resistance: Recent Advances and Future Perspectives

**DOI:** 10.3390/ijms27167187

**Published:** 2026-08-11

**Authors:** Jialong Xie, Xingxuan Ren, Zhibin Wang, Weidong Xie

**Affiliations:** 1 College of Life Sciences and Oceanography, Shenzhen University, Shenzhen 518055, China; suat25060190@stu.suat-sz.edu.cn; 2 Department of Biopharmaceutical Sciences, Faculty of Pharmaceutical Sciences, Shenzhen University of Advanced Technology, Shenzhen 518107, China; 3 Center for Cancer Immunotherapy, Institute of Biomedicine and Biotechnology, Shenzhen Institutes of Advanced Technology, Chinese Academy of Sciences, Shenzhen 518055, China; 4 Shenzhen Key Laboratory of Health Science and Technology, Institute of Biopharmaceutical and Health Engineering, Shenzhen International Graduate School, Tsinghua University, Shenzhen 518055, China; rxx25@mails.tsinghua.edu.cn; 5 Open FIESTA (Faculty for Innovation, Education, Science, Technology and Art) Center, Shenzhen International Graduate School, Tsinghua University, Shenzhen 518055, China; 6 State Key Laboratory of Chemical Oncogenomics, Shenzhen International Graduate School, Tsinghua University, Shenzhen 518055, China

**Keywords:** cancer, drug resistance, paclitaxel

## Abstract

Paclitaxel (PTX) is a core first-line chemotherapeutic agent used to treat a broad spectrum of cancers, which remain leading causes of death worldwide. However, the emergence of intrinsic and acquired resistance critically contributes to chemotherapy failure, thereby promoting tumor recurrence and metastasis and resulting in a poor prognosis. PTX resistance is a complex biological process driven by multiple factors and cross-regulated signaling pathways. It encompasses a wide variety of mechanisms, including aberrations in microtubules and related proteins; changes in mitotic systems and chromosomal instability (CIN); epigenetic modifications including ncRNA regulation, DNA methylation, and histone modification; enhanced drug efflux and dysregulation of proliferation and apoptotic signaling pathways; enhanced protective autophagy; metabolic reprogramming; cell plasticity (increased EMT and cancer cell stemness); TME remodeling; and immune regulation. This review systematically examines the core molecular mechanisms of PTX resistance, summarizes cutting-edge strategies to reverse chemoresistance and their clinical translation, and discusses current challenges and future directions, thereby providing a theoretical framework for optimizing PTX-based regimens and overcoming clinical resistance.

## 1. Introduction

### 1.1. Current Status of Cancer Epidemiology and Challenges in Clinical Diagnosis and Treatment

Cancer remains a leading cause of death worldwide and a major global health challenge, with persistently high incidence rates [1]. Lung cancer, breast cancer, and colorectal cancer are among the most common malignancies in the world [2,3,4]. Cancer treatment primarily includes surgery, radiation therapy, and drug therapy. Surgery is generally restricted to early-stage cancer, and radiation therapy often causes significant side effects; consequently, systemic drug therapy constitutes the mainstay of treatment for the majority of patients [5]. In recent years, various forms of systemic therapy have advanced rapidly, including classical chemotherapy, targeted therapy, immunotherapy, and epigenetic drugs, including antibody–drug conjugates (ADCs) and proteolysis-targeting chimeras (PROTACs) [6,7,8,9,10,11]. Although chemotherapy regimens are well established, they are frequently associated with severe adverse effects—such as myelosuppression, gastrointestinal toxicities, and alopecia—leading to poor patient compliance. Moreover, resistance to chemotherapy often emerges during treatment [11]. Newer modalities such as targeted therapy are generally associated with milder side-effect profiles; however, resistance to these agents also frequently develops, and they remain relatively costly. In advanced-stage disease, resistance to targeted therapies is prevalent, and widespread systemic metastasis is common. Current management therefore relies heavily on classical chemotherapeutic agents, including both cell-cycle-specific and non-specific drugs. However, drug resistance remains a critical obstacle for these agents, as exemplified by paclitaxel, a first-line chemotherapeutic drug that is frequently compromised by the emergence of resistance [12,13,14,15].

### 1.2. Clinical Challenges in the Use of PTX and Drug Resistance

**Figure 1:**
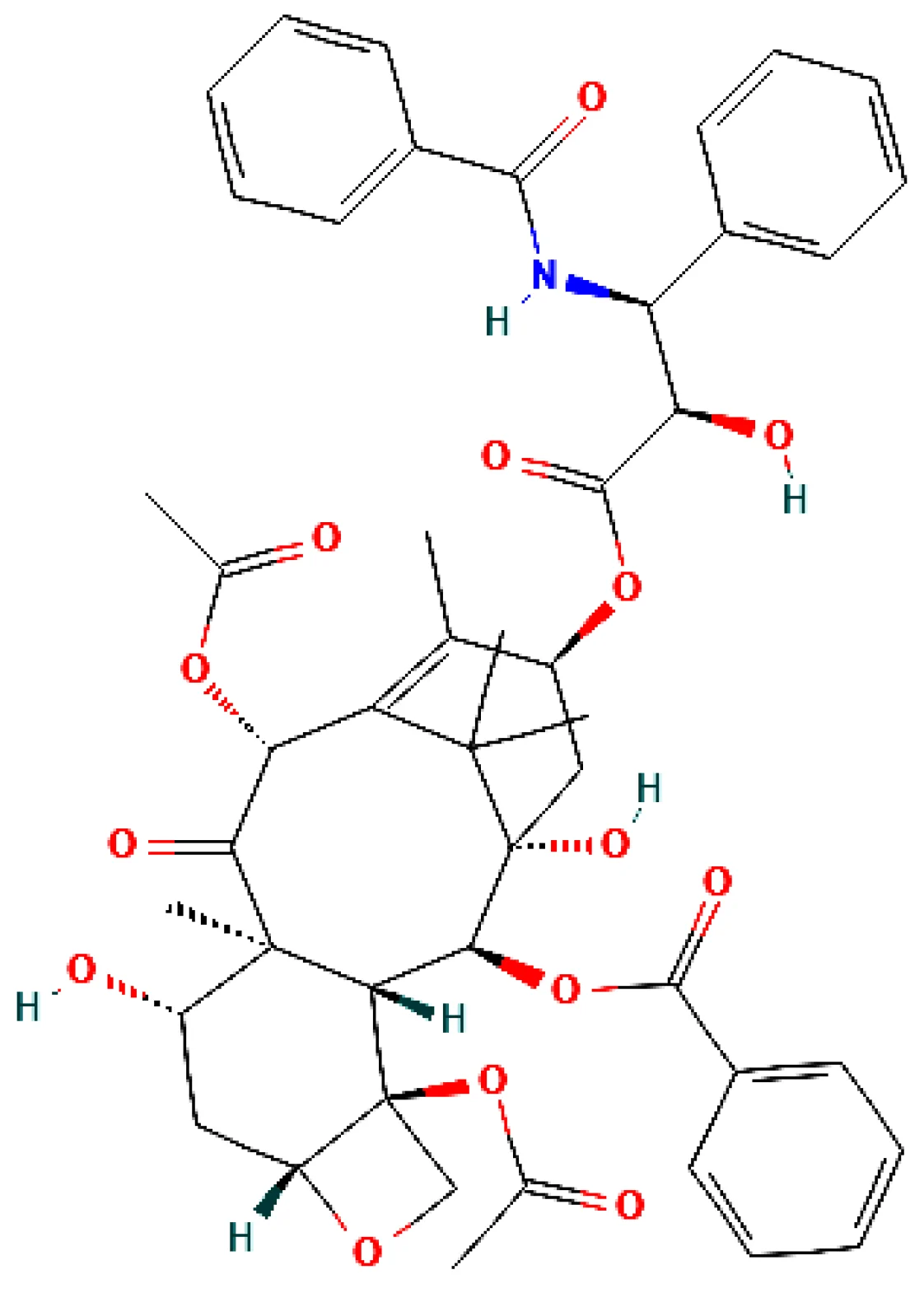
Molecular structure of PTX (https://pubchem.ncbi.nlm.nih.gov/compound/36314, accessed on 31 May 2024).

PTX (trade name Taxol^®^), a natural anticancer compound originally isolated from the bark of the yew tree, is currently used as a first-line chemotherapeutic agent for a wide range of cancers, including ovarian cancer, breast cancer, non-small-cell lung cancer, cervical cancer, head and neck squamous cell carcinoma, esophageal cancer, and gastric cancer [16] (Figure 1). PTX exerts anticancer effects by promoting microtubule assembly [17]. However, resistance to PTX frequently emerges during clinical use, with declining efficacy often observed after multiple treatment cycles [13]. PTX resistance may share common and cancer-type-specific mechanisms in different heterogeneous cancers. However, the underlying mechanisms are complex and remain to be fully elucidated, resulting in a lack of effective strategies to overcome this challenge in the clinic.

### 1.3. Purpose, Content, and Method of This Review

A comprehensive understanding of these mechanisms is therefore critical for both basic research and clinical practice. A previous review introduced the mechanisms of resistance to PTX treatment and discussed related tactics in breast cancer [18]. However, PTX is widely used in various heterogeneous cancers, and PTX resistance is a complex biological process involving the cross-regulation of multiple factors and pathways. The exact molecular mechanisms of PTX resistance are still poorly understood and need further evaluation.

Therefore, this review systematically examines the core molecular mechanisms of PTX resistance, summarizes cutting-edge strategies to reverse chemoresistance and their progress toward clinical translation, and discusses existing challenges and future directions, thereby providing a theoretical framework for optimizing PTX-based regimens and overcoming clinical resistance.

We searched databases including PubMed, Web of Science, Wiley Online Library, Oxford Academic Journals, Scopus, and CNKI. The primary literature search focused on the period from 2020 to 2026; however, older foundational studies that provided essential background or established key mechanistic concepts were also included. Subject search terms were combinations of “cancer OR carcinoma OR tumor OR malignancy”, “paclitaxel OR taxol”, and “drug resistance OR multidrug resistance OR drug resist OR chemoresist OR paclitaxel resist OR taxol resist”. Additional keywords were employed for each section according to its specific content. The literature search was conducted by two independent individuals.

## 2. The Antitumor Effects and Molecular Mechanisms of PTX

### 2.1. Molecular Targets of PTX: Structural and Kinetic Basis of the Microtubule System and Disruption of the Mitosis Process

PTX is a classic microtubule-targeting agent (MTA). Microtubules are essential components of the cytoskeleton and the mitotic apparatus. They are assembled from α-tubulin and β-tubulin heterodimers and microtubule-associated proteins (MAPs) [19,20]. They form the mitotic spindle during cell division and play crucial roles in maintaining cell shape, motility, and intracellular transport [13]. PTX binds to the taxane-binding pocket located on β-tubulin, disrupting microtubule dynamics by stabilizing microtubules and inducing their bundling [19,21]. As a consequence, PTX potently disrupts mitotic progression and leads to cell cycle arrest at the G2/M phase [13,22,23], ultimately triggering apoptotic cell death [23,24,25].

### 2.2. PTX-Induced Apoptosis Signaling Pathways and Mechanisms

**Figure 2:**
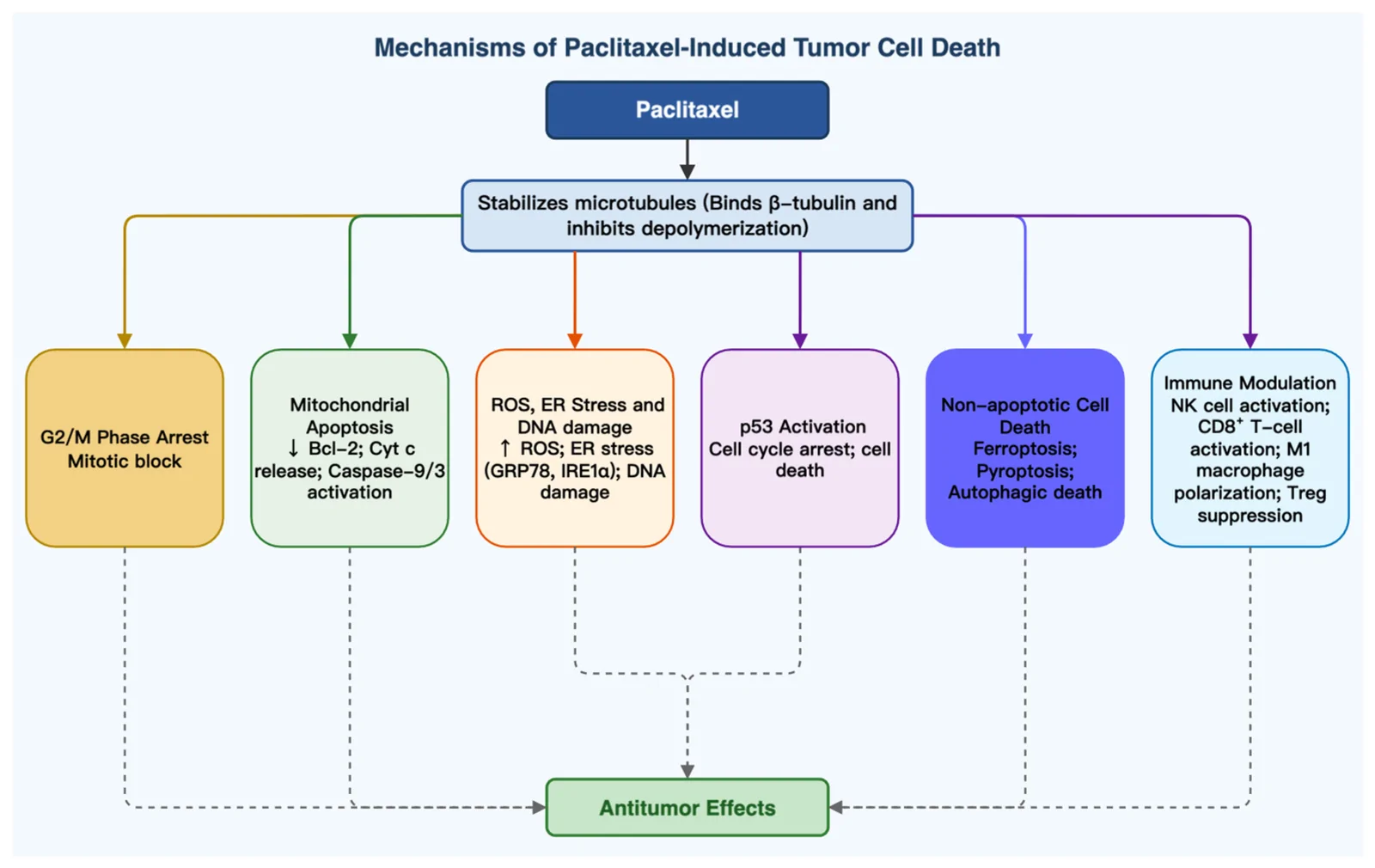
Schematic diagram of the mechanism of PTX-mediated tumor cell death. **↓ and ↑ indicate downregulation and upregulation of the indicated molecules/processes, respectively**.

As described above, PTX inhibits mitosis to suppress cell proliferation while simultaneously activating intracellular apoptotic pathways [13] (Figure 2). Induction of apoptosis is a primary mechanism of PTX in cancer cells, involving the regulation of mitochondrial function, production of reactive oxygen species (ROS), cell cycle modulation, and activation of antitumor immunity [17,26].

#### 2.2.1. Mitochondrial Apoptosis Pathway

Apoptosis is a form of programmed cell death in which mitochondria play a critical role. Studies have shown that PTX acts on mitochondria and inhibits the anti-apoptotic protein B-cell leukemia-2 (Bcl-2) [26,27]. PTX triggers the release of cytochrome c (Cyt c) from mitochondria in human neuroblastoma cells, leading to apoptosis [28]. During PTX-induced apoptosis, Caspase-3 and Caspase-8 mediate a mitochondrial amplification loop that is essential for optimal Cyt c release, opening of the mitochondrial permeabilization transition pore (mPTP), and cell death [29].

#### 2.2.2. ROS, Endoplasmic Reticulum (ER) Stress, and DNA Damage Pathways

Oxidative stress has a significant impact on cancer cell survival through mitochondrial apoptosis, ER stress, and DNA damage pathways. Sometimes, these pathways can interact with each other. PTX induces the production of ROS in the choriocarcinoma cell lines JEG-3 and BEWO and increases the Bax/Bcl-2 ratio [30]. In osteosarcoma cells, PTX inhibits HOS-732 cell proliferation while elevating ROS levels and ER stress responses, inducing the expression of ER stress-related genes and proteins, including DDIT3 mRNA, and the proteins glucose-regulated protein 78 (GRP78), spliced X-box binding protein 1 (XBP-1s), and inositol-requiring enzyme 1α (IRE1α), which promotes apoptosis [31]. In ovarian cancer cells, PTX activates both the IRE-1 and PERK kinases and induces ER stress and causes cell death through WWOX [32]. PTX increases ROS production in non-small-cell lung cancer (NSCLC) cells (PC9-MET), leading to DNA damage [33].

#### 2.2.3. Activation of the p53 Transcription Factor

The transcription factor p53 is a key tumor suppressor that regulates various cellular responses, including cell cycle arrest, apoptosis, and DNA repair, to prevent the development of cancer [34]. Beyond inducing cell death, p53 can also block cell proliferation by halting the cell cycle at specific checkpoints. In some studies, p53 was found to mediate PTX-induced cancer cell death, and PTX has shown more sensitivity to p53 activation [35,36]. PTX significantly inhibits the proliferation of A549 cells and induces cell death through upregulation of maternal expression gene 3 (MEG3) and increased p53 expression [37]. PTX can induce apoptosis via two distinct pathways through activation of the tumor suppressor protein p53, depending on the drug concentration [38]. Following PTX treatment, the threonine 9 (Thr9) site of PDZ-binding kinase (PBK) is phosphorylated, and p53 expression is upregulated [38]. Importantly, TP53 is among the most frequently mutated genes in human cancers, and PTX can still induce cell death through p53-independent mechanisms in tumors harboring dysfunctional or mutant TP53. These mechanisms include mitotic catastrophe or direct disruption of mitochondrial function and ROS-mediated apoptosis. Therefore, while p53 activation contributes to PTX-induced apoptosis in some cellular contexts, it should not be regarded as a universal mediator of PTX cytotoxicity.

### 2.3. The Effects of PTX on Non-Apoptotic Cell Death

Ferroptosis, pyroptosis, and autophagy-mediated cell death have been closely linked to cancer cell survival. PTX has been shown to modulate all of these death processes.

#### 2.3.1. PTX Can Induce Ferroptosis in Cancer Cells

Ferroptosis is an iron-dependent form of non-apoptotic cell death [17]. Its occurrence is associated with elevated levels of ROS resulting from the loss of glutathione peroxidase 4 (GPX4) activity [39]. Non-apoptotic forms of cell death may contribute to the selective elimination of certain tumor cells or be activated under specific pathological conditions [40]. Studies have shown that PTX enhances oxidative stress in KTC-1 cells, thereby inducing ferroptosis and inhibiting thyroid cancer [41]. The anticancer effects of PTX are associated with the induction of ferroptosis in triple-negative breast cancer (TNBC), which is regulated by ferritin autophagy mediated by nuclear receptor coactivator 4 (NCOA4) [42].

#### 2.3.2. PTX Can Induce Pyroptosis in Cancer Cells

Pyroptosis is an inflammatory form of cell death associated with the activation of inflammasomes and the release of pro-inflammatory cytokines such as interleukin-1 (IL-1) and interleukin-18 (IL-18) [17]. It can also enhance antitumor immunity to mediate tumor suppression. Promoting pyroptosis in cancer cells is an effective strategy for tumor suppression. Studies have shown that PTX induces pyroptosis via two pathways—Caspase 1/GSDMD and Caspase 3/GSDME—accompanied by maturation of interleukin-1β (IL-1β) [17,43,44]. For example, in nasopharyngeal carcinoma cells, PTX-induced pyroptosis is mediated through the caspase-1/GSDMD pathway [44]. In breast cancer MCF-7 cells, PTX was shown to promote GSDME expression through reversing DNA demethylation [45]. In ovarian cancer cells, PTX induces pyroptosis by inhibiting the volume-sensitive chloride channel. Therefore, it is likely that PTX induces pyroptosis in cancer cells through multiple pathways.

#### 2.3.3. PTX Can Modulate Autophagy to Influence Cancer Cell Fate

Autophagy is a fundamental mechanism for maintaining cellular homeostasis that is regulated by more than 30 genes [46]. In cancer, autophagy acts as a double-edged sword: moderate or transient autophagy frequently serves a cytoprotective function by recycling damaged organelles and metabolic substrates, whereas excessive or sustained autophagy can promote cell death [47]. The functional outcome of autophagy in PTX-treated cells depends heavily on autophagic flux—the complete process from autophagosome formation to lysosomal degradation—rather than on static measurements of autophagy markers alone. For instance, an increase in LC3-II levels may indicate enhanced autophagosome formation or, conversely, impaired autophagosome clearance due to lysosomal dysfunction [48]; therefore, assessing autophagic flux using lysosomal inhibitors is essential to accurately interpreting autophagy activity.

In the context of PTX treatment, both cytoprotective and cytotoxic functions of autophagy have been documented [17,47]. PTX has been shown to increase levels of microtubule-associated protein 1 light chain 3-II (LC3-II) [48], which is accompanied by inhibition of proliferation in gastric cancer cells [49,50]. Notably, while PTX induces apoptosis, autophagy is concomitantly activated, and inhibition of autophagy can enhance the pro-apoptotic effects of PTX, suggesting that the autophagy induced under these conditions may serve a pro-survival role [51]. Conversely, under certain circumstances, PTX-induced autophagy can mediate cell death in AGS cells [52]. Thus, PTX does not universally induce “autophagic cell death”; rather, it engages the autophagy machinery in a context-dependent manner that can either protect cancer cells from or contribute to their demise.

### 2.4. The Effects of PTX on Immune Regulation

The immune system plays a crucial regulatory role in tumor cell growth, and PTX has been shown to activate the immune system in vivo [17]. It exerts immunostimulatory effects against tumors and can also regulate lymphocyte activation [53]. It has been shown to reduce the number and suppressive function of regulatory T cells (Tregs) in mice [54] and to modulate the TLR4 inflammatory pathway and activate antitumor immune responses by reprogramming macrophages into the M1 phenotype [17,55]. Clinical studies have demonstrated that PTX treatment induces antitumor activity in natural killer (NK) cells and CD8^+^ T lymphocytes in patients with human epidermal growth factor receptor 2 (HER2)-positive breast cancer [17,56].

However, the net immunological effect of PTX is highly context-dependent and influenced by dose, formulation, tumor type, and the biological milieu. While the antitumor immune activation described above has been observed under specific experimental and clinical conditions, PTX can also exert opposing effects through the same TLR4 pathway. Specifically, PTX-mediated TLR4 activation in tumor cells can trigger systemic inflammatory responses that promote angiogenesis and de novo lymphangiogenesis within the tumor microenvironment, thereby facilitating tumor metastasis [57]. Moreover, in the resistant setting, the immunosuppressive remodeling of the TME—characterized by an increase in Tregs and the polarization of tumor-associated macrophages toward the M2 phenotype—can dampen antitumor immunity and support the survival of drug-resistant tumor cells [55,58]. Therefore, the immunomodulatory properties of PTX should be viewed as a double-edged sword, capable of both enhancing antitumor immunity and, under certain conditions, contributing to an immunosuppressive or pro-metastatic milieu.

## 3. The Core Molecular Mechanisms of PTX Resistance

**Figure 3:**
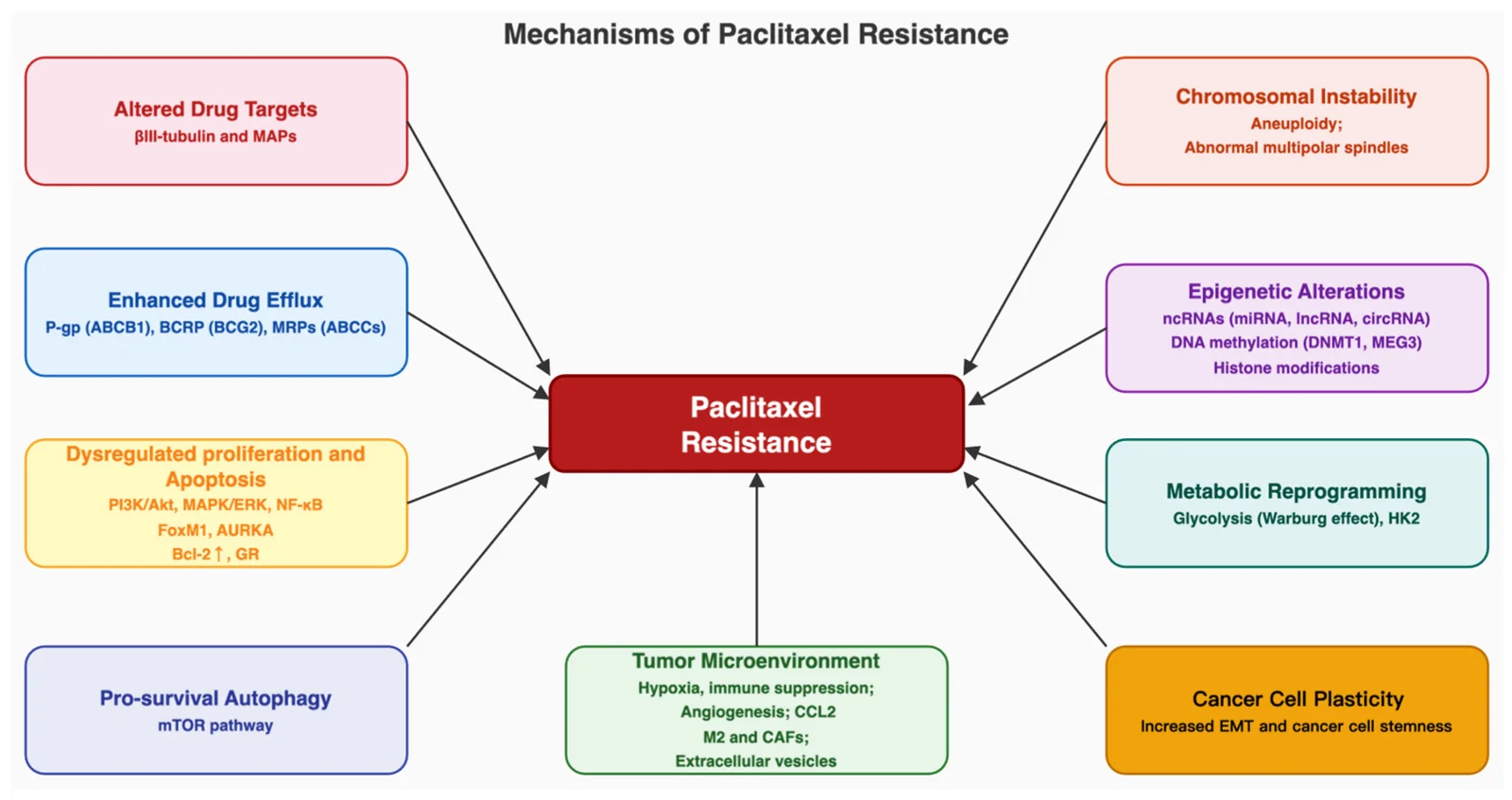
Diagram of the mechanism of PTX resistance in tumor cells. **↑ indicates upregulation of the indicated molecules/processes**.

Although PTX plays a crucial role in chemotherapy and has a unique mechanism of action, resistance frequently emerges during treatment, leading to reduced efficacy or increased side effects. Understanding the mechanisms of resistance is therefore of significant research importance. These mechanisms are highly complex, involving multiple molecular and cellular biological processes. The mechanisms of PTX resistance usually encompass structural and functional abnormalities in drug targets, enhanced drug efflux, dysregulation of proliferation and apoptotic pathways, induced protective autophagy, metabolic reprogramming, epigenetic modifications, chromosomal instability, TME remodeling, and cell plasticity (increased EMT and cancer cell stemness) (see Figure 3).

### 3.1. Abnormal Drug Targets: Alterations in the Microtubule System Mediate Drug Resistance

#### 3.1.1. An Overview of the Cytoskeletal Microtubule and Mitotic Systems

In eukaryotes, microtubules are essential for diverse cellular processes, including mitosis and meiosis, cell motility, maintenance of cell shape, and intracellular transport of macromolecules and organelles. They are primarily composed of polymers formed by the self-assembly of α- and β-tubulin heterodimers [20,59]. Mutations in beta-tubulin or abnormal expression of specific β-tubulin isoforms (particularly βIII-tubulin) or microtubule-regulating proteins have been clinically associated with tumor aggressiveness and chemoresistance [19,20]. Microtubule-associated proteins (MAPs) regulate microtubule stability and spindle formation during cell division.

#### 3.1.2. Mechanisms by Which Microtubule and Mitotic Systems Mediate PTX Resistance

One study showed that upregulation of β-tubulin may lead to PTX resistance in advanced ovarian cancer [60]. PTX-resistant cells display increased microtubule dynamics. Focal adhesion kinase (FAK) is overexpressed and persistently activated in most solid tumors. FAK and microtubules exhibit reciprocal crosstalk regulation; in particular, upregulation of FAK enhances microtubule dynamics in ovarian cancer cells, thereby mediating resistance to PTX [61].

Beyond tubulin isotype alterations, changes in microtubule-associated proteins (MAPs) also contribute to PTX resistance. Changes in the expression of MAPs such as stathmin, tau, and MAP4 contribute to PTX resistance. The microtubule-destabilizing protein stathmin 1 is a direct transcriptional target of FOXM1; its upregulation counteracts the microtubule-stabilizing effect of PTX by promoting microtubule depolymerization [62]. More broadly, alterations in the expression or post-translational modifications of MAPs that stabilize or destabilize microtubules—such as MAP4 and Tau—can shift the equilibrium of microtubule dynamics, thereby influencing the net sensitivity of cancer cells to microtubule-targeting agents [20].

Altered spindle assembly checkpoint proteins, mitotic checkpoint adaptation, and mitotic slippage are usually associated with PTX resistance. After binding to microtubule proteins, PTX may cause them to become dysfunctional and thereby activate the spindle assembly checkpoint, causing mitotic arrest and slippage, which may cause cancer cells to become tetraploid or polyploid cells. The genomic instability in polyploid cells ultimately leads to apoptosis or senescence. Particularly, mitotic slippage drives cells to exit mitosis prematurely without chromosome segregation or cytokinesis, enter a pseudo-G1 phase, form 4N tetraploid/polyploid cells, and evade mitotic cell death.

### 3.2. Mechanisms of PTX Multidrug Resistance (MDR) Mediated by Classic Drug Efflux Pumps

#### 3.2.1. Structural and Functional Basis of the ATP-Binding Cassette (ABC) Transporter

ABC transporters mediate the active efflux of a wide variety of compounds—regardless of their structure or mechanism of action—across cellular and organelle membranes [63]. In cancer, ABC transporters play a critical role in the multifaceted mechanisms underlying MDR by effluxing chemotherapeutic agents from cells [63,64]. MDR remains a major obstacle in cancer therapy and is typically mediated by the overexpression of ABC transporters, such as P-glycoprotein (P-gp) and breast cancer resistance protein (BCRP) [63,65].

#### 3.2.2. Core ABC Transporter Subtypes and Their Functions in Mediating PTX Resistance

ABC transporters have many subtypes, among which ABCB1 (P-gp) is the most classic efflux pump associated with PTX resistance. P-gp is encoded by the MDR1/ABCB1 gene and normally mediates the efflux of toxins from normal cells while also conferring resistance to certain chemotherapeutic agents [66]. Using energy from ATP hydrolysis, P-gp effluxes a broad range of drugs from the cytoplasm of cancer cells, including anthracyclines, vinca alkaloids, taxanes, kinase inhibitors, isomerase inhibitors, polymerase inhibitors, and proteasome inhibitors. When overexpressed, P-gp renders many therapeutic agents ineffective, ultimately leading to drug resistance [67,68].

ABCG2, also known as breast cancer resistance protein (BCRP), is another ABC transporter implicated in PTX resistance. As shown by Kohli et al., ABCG2 can be upregulated and contributed to the resistant phenotype [69]. In contrast to ABCB1 and ABCG2, which primarily function through drug efflux at the plasma membrane, some ABCC subfamily members (ABCC3-, ABCC5-, and ABCC10) confer resistance even through lysosomal sequestration of the drug, representing a mechanistically distinct mode of action [70]. Recent studies have confirmed that several members of the ABCC subfamily differ significantly in their mechanisms of action from P-gp [71].

Taken together, these findings highlight a network of ABC transporters with differing mechanisms, wherein ABCB1 remains the most extensively validated efflux pump in clinical PTX resistance, while ABCG2 and ABCC members contribute through complementary or distinct routes.

### 3.3. Abnormalities in Cell Proliferation and Apoptosis Pathways and Factors Lead to the Development of Drug Resistance

During PTX treatment, pro-proliferative and anti-apoptotic signaling pathways become dysregulated, contributing to the development of drug resistance. Tumor cells mediate PTX resistance either by preventing PTX-induced mitotic arrest from effectively triggering apoptosis or by directly regulating microtubule dynamics, through the activation of the phosphoinositide 3-kinase/protein kinase B (PI3K/Akt) pathway, the mitogen-activated protein kinase/extracellular signal-regulated kinase (MAPK/ERK) pathway, the nuclear factor κB (NF-κB) pathway, forkhead box M1 (FoxM1), Aurora kinase A (AURKA), and Bcl-2.

#### 3.3.1. Abnormalities in the PI3K/Akt Pathway

(1) The Pro-proliferative and Anti-apoptotic Roles of PI3K/Akt in Tumors

The phosphoinositide 3-kinase (PI3K)/protein kinase B (AKT) pathway regulates cell division, growth, and survival [72]. As a lipid kinase, phosphoinositide 3-kinase plays a critical role in cell proliferation, cell survival, cell cycle progression, migration, and invasion [73,74,75]. More importantly, the PI3K/Akt signaling pathway is activated in multiple cancers, including multidrug-resistant tumors, and is directly involved in regulating growth-related transcription factors that induce cancer stem cell (CSC) and epithelial–mesenchymal transition (EMT) phenotypes [73,75,76].

(2) Abnormal activation of the PI3K/Akt pathway mediates PTX resistance

Abnormal activation of the PI3K/Akt pathway is a key molecular mechanism underlying acquired resistance to PTX in various tumors [75,77]. The C-X-C chemokine receptor 4 (CXCR4)-PI3K/Akt/mammalian target of rapamycin (mTOR) axis promotes PTX resistance by inducing the formation of cancer stem cells and EMT [75]. The PI3K/Akt pathway further contributes to resistance by inhibiting pro-apoptotic signals, inhibiting FOXO3-mediated cell cycle arrest, and enhancing the expression of efflux transporters [78,79,80]. Tenascin C (TNC) promotes glioma cell proliferation and stemness by regulating the PI3K/AKT signaling pathway, thereby inhibiting chemoresponsiveness to PTX [81]. MNX1-AS1 promotes breast cancer tumor progression and induces PTX resistance by activating the integrin α6 (ITGA6)/PI3K/AKT pathway [72].

#### 3.3.2. Abnormalities in the MAPK/ERK Pathway

(1) The MAPK/ERK pathway and its role in tumors

The MAPK/ERK cascade is a key signaling pathway involved in cell growth, differentiation, and survival [82]. The mitogen-activated protein kinase (MAPK) cascade is a highly conserved signaling complex that translates extracellular stimuli into various cellular responses [83,84]. The extracellular signal-regulated kinase 1/2 (ERK1/2) pathway is primarily activated by mitogens; it activates pro-survival pathways to promote cell proliferation and migration while also regulating apoptosis, differentiation, and senescence. This pathway is frequently upregulated in many human tumors [84,85].

(2) Mechanisms of PTX Resistance Involving the MAPK/ERK Pathway

PTX resistance in tumors is associated with the MAPK/ERK pathway [86,87]. PTX has been shown to activate ERK in cervical cancer cells, thereby promoting drug resistance [88]. PTX induces sustained activation of the rat sarcoma oncogene homolog (Ras)/MAPK/ERK kinase (MEK)/ERK pathway in human esophageal squamous cell carcinoma cells, thereby inhibiting apoptosis [82]. The level of phosphorylated ERK (p-ERK) is significantly upregulated in A549/PTX-resistant cells, contributing to cell survival and proliferation and ultimately leading to drug resistance [89]. The CD44v10 adhesion molecule upregulates glucose transporter 1 (GLUT1) via activation of the MAPK/ERK signaling pathway, thereby promoting glycolysis and inducing PTX resistance [90].

#### 3.3.3. Abnormalities in the NF-κB Pathway

(1) Overview of the NF-κB Pathway and Its Role in Cancer

NF-κB is a family of transcription factors that plays a key role in various physiological and pathological processes [91]. NF-κB is a sequence-specific transcription factor that functions as a transcriptional regulator composed of distinct protein dimers, binding to a conserved sequence motif known as the κB site, and is involved in inflammatory and innate immune responses [92]. Its activation regulates the expression of numerous gene products and is associated with various cancer-related cellular processes, including inflammation, cellular transformation, proliferation, angiogenesis, invasion, metastasis, and resistance to chemotherapy and radiation therapy [93].

(2) Mechanisms by Which the NF-κB Pathway Mediates PTX Resistance

The NF-κB pathway promotes the enrichment of tumor stem cells and can be activated by PTX, thereby leading to PTX resistance [94]. NF-κB p65 is essential for late-stage tumorigenesis and mediates and sustains the epithelial–mesenchymal transition (EMT) phenotype [95]. Many additional factors can regulate the NF-κB pathway, and PTX resistance may be related to these. In breast cancer, overexpression of T-cell immunoglobulin and mucin 3 (Tim-3) activates the NF-κB pathway, thereby driving PTX resistance [96]. TAOK3 increases breast cancer cell resistance to PTX by upregulating the NF-κB signaling pathway [97].

#### 3.3.4. The Role of FoxM1 in Promoting Drug Resistance

(1) The FoxM1 Transcription Factor and Its Pro-proliferative and Anti-apoptotic Effects in Tumors

Forkhead box M1 (FOXM1), a member of the forkhead box transcription factor family, possesses a conserved wing-and-helix DNA-binding domain and is a key transcription factor (TF) that regulates cell proliferation and exhibits cell-cycle-specific expression patterns [62,98,99]. This factor controls the transcription of multiple cell-cycle-related genes and regulates various critical biological processes to ensure the accurate progression of DNA replication and mitosis, thereby playing an active role in cell proliferation, migration, angiogenesis, stem cell regeneration, DNA damage repair, apoptosis, and inflammatory responses [98,100,101]. FOXM1 is highly expressed in various malignant tumors and exerts biological effects through multiple molecular mechanisms, thereby promoting the proliferation of malignant cells in different cancer types [98].

(2) FOXM1-Mediated PTX Resistance: Multifactorial Regulatory Mechanisms

FOXM1 can directly upregulate the expression of the microtubule-disassembling protein stathmin 1 to antagonize the effects of PTX and regulate threonine-tyrosine kinase (TTK), thereby reducing tumor sensitivity to PTX [62,102]. FOXM1 promotes lipid droplet accumulation by activating phospholipase D1 (PLD1), thereby driving PTX resistance [103]. Acylglycerol kinase (AGK) activates the Janus kinase 2 (JAK2)/signal transduction and transcription activator 3 (STAT3) signaling pathway, thereby promoting FOXM1 transcription and ultimately enhancing PTX resistance in nasopharyngeal carcinoma cells [104]. FOXM1 promotes resistance in cervical cancer cells by regulating the transcription of the ATP-binding cassette C subfamily member 5 (ABCC5) gene [105].

#### 3.3.5. The Role of Aurora Kinase A in Promoting Drug Resistance

(1) The Pro-proliferative and Anti-apoptotic Effects of AURKA in Tumors

Aurora kinases (AKs) are a family of serine/threonine kinases essential for mitosis; in mammals, this family comprises three members: Aurora kinase A (AK-A), Aurora kinase B (AK-B), and Aurora kinase C (AK-C) [106,107]. AK-A is overexpressed in various cancers, including breast, lung, bladder, and pancreatic cancers [107].

(2) Mechanisms by which AURKA mediates PTX resistance

Studies have shown that amplification of Aurora kinase A (AK-A) bypasses the mitotic spindle assembly checkpoint and induces resistance to taxane drugs [106,108]. Mechanistically, this can lead to mitotic slippage, a process in which cells arrested in mitosis by PTX eventually exit mitosis without undergoing proper chromosome segregation or cytokinesis, surviving as tetraploid or polyploid cells rather than undergoing apoptosis. Mitotic slippage thus represents an alternative cell fate that directly antagonizes the cytotoxic intent of microtubule-targeting agents. Aurora A may regulate P-gp through phosphorylation of ERK2 (p-ERK2), thereby contributing to PTX resistance in breast cancer cells [109]. PTX treatment enhances AURKA protein stability, thereby creating a positive feedback loop for acquired resistance [110]. Overexpression of Aurora kinase in triple-negative breast cancer cells leads to PTX resistance and attenuates chemotherapy-induced apoptosis [110].

#### 3.3.6. The Role of the BCL-2 Family in Promoting Resistance to PTX

(1) Overview of the BCL-2 Family and Its Role in Tumors

Proteins in the B-cell lymphoma-2 (BCL-2) family regulate programmed cell death, with some members (such as BCL-2 and BCL-XL) inhibiting apoptosis and others (such as the BCL-2-associated X protein BAX and the BCL-2 antagonist/killer factor BAK) promoting cell death, thereby modulating the cellular response to various anticancer drugs [111,112]. Bcl-2 inhibits cell death by binding to the BCL-2 homology domain 3 (BH3) of pro-apoptotic Bcl-2 family proteins, thereby preventing the transition of mitochondrial outer membrane permeability, a process considered a critical irreversible step in apoptosis [113].

(2) Mechanism of BCL-2-Mediated PTX Resistance

Bcl-2 plays a critical role in tumorigenesis and chemoresistance, and its oncogenic function is directly linked to PTX resistance [113]. Compared to PTX-sensitive parental cells, PTX-resistant ovarian cancer cells exhibit higher expression levels of various pro-survival Bcl-2 family members, including BCL-2, BCL-XL, and myeloid cell leukemia 1 (MCL-1) [114]. Following knockdown of FK506-binding protein 38 (FKBP38), Bcl-2 loses the ability to bind to the apoptosis-inducing factor Bcl-2-associated death promoter (Bad). Without the inhibitory effect of Bad, Bcl-2 fully exerts its anti-apoptotic capacity, thereby enhancing the resistance of esophageal cancer cells to PTX [115]. Targeting the Bcl-2 family can reverse the resistance of PTX cells [116]. Runt-related transcription factor 1 (RUNX1) promotes PTX resistance by upregulating BCL2 [117].

#### 3.3.7. The Role of Glucocorticoid Receptor Signaling in Promoting Resistance to PTX

(1) Overview of the glucocorticoid receptor and its role in tumors

The glucocorticoid receptor (GR), encoded by the *NR3C1* gene, is a ubiquitously expressed, ligand-activated transcription factor that mediates the effects of glucocorticoid hormones [118,119]. Beyond its classical genomic actions, GR can engage in rapid non-genomic signaling pathways [119]. High GR expression has been clinically associated with poor prognosis and impaired chemotherapy effectiveness, as GR activation can induce pro-survival pathways and cell differentiation [119,120]. It has also been shown to act either as an oncogene or an oncosuppressor depending on the tumor histotype, and its activity has been explored in several solid tumors [121].

(2) Mechanisms by which GR signaling mediates PTX resistance

GR activation has been shown to impair chemotherapy effectiveness by modulating apoptotic pathways [122,123]. Cortisol, the endogenous GR ligand, can blunt PTX-induced apoptosis in cancer cells, and this effect is reversible by GR antagonists such as relacorilant [124]. Mechanistically, GR activation promotes pro-survival gene expression programs that directly antagonize the apoptotic effects of PTX [120]. Psychological stress-induced GR activation drives epithelial–mesenchymal transition (EMT) and metastasis by transcriptionally upregulating NUPR1, which subsequently increases the expression of SNAI2 (SLUG), a master regulator of EMT [119,125]. GR signaling also shapes an immunosuppressive tumor microenvironment by promoting the recruitment of polymorphonuclear myeloid-derived suppressor cells (PMN-MDSCs) and enhancing regulatory T-cell-mediated immune suppression, thereby facilitating immune evasion and indirectly contributing to chemoresistance [126,127,128].

### 3.4. Autophagy-Mediated PTX Resistance

#### 3.4.1. The Role of Autophagy in Tumors

Autophagy is an intracellular degradation pathway that transports cytoplasmic components to lysosomes for degradation and recycling [129]. In advanced cancer, autophagy generates energy by degrading redundant organelles and proteins, thereby promoting tumor survival under oxidative stress and nutrient deprivation [129,130]. In early-stage tumors, autophagy acts as a tumor suppressor by clearing cytotoxic proteins and damaged organelles, thereby maintaining cellular homeostasis and genomic stability while also regulating cell death and senescence [129,131,132].

#### 3.4.2. Mechanisms of PTX Resistance Mediated by Autophagy

The role of autophagy in the antitumor effects of PTX remains controversial. Some studies confirm that autophagy is one of the mechanisms by which PTX kills tumor cells, while others indicate that autophagy acts as a pro-survival mechanism in tumor cells, mediating drug resistance [129,133,134]. Increasing evidence suggests that in established resistance, tumor cells predominantly exploit the pro-survival functions of autophagy to mediate resistance. Tumor cells employ autophagy to withstand adverse microenvironmental conditions, thereby driving the resistance phenotype in PTX resistance through multiple mechanisms [135]. Drugs targeting the PI3K/AKT/mammalian target of rapamycin complex 1 (mTORC1) pathway can induce autophagy by inhibiting mTORC1, which may, in turn, mediate the development of resistance [136]. The regulation of autophagy by PTX is both time- and dose-dependent: protective autophagy induced after PTX administration mediates resistance, whereas pre-induced excessive autophagy can trigger autophagic cell death [137].

The ultimate outcome of autophagy in the context of PTX treatment—whether it favors cell survival or cell death—is determined by multiple contextual factors. The intensity and duration of autophagic flux appear to be critical: moderate or transient autophagy induced by PTX frequently serves a cytoprotective function, enabling the clearance of damaged organelles and the recycling of metabolic substrates, whereas sustained or excessive autophagy can progress to autophagy-dependent cell death, a process that is mechanistically distinct from apoptosis and requires rigorous evidence that cell death is actually executed by autophagy rather than merely accompanied by it. In addition, the basal metabolic and genetic landscape of the cancer cell, including the status of key regulators such as p53, PI3K/Akt/mTOR axis activity, and the expression levels of autophagy-related (ATG) proteins, influences the threshold at which autophagy shifts from a survival mechanism to a lethal process. Furthermore, crosstalk between autophagy and apoptosis—mediated by factors such as the Bcl-2 family proteins that regulate both pathways—can dictate the cellular decision. Thus, the dual role of autophagy in PTX resistance is not an intrinsic property of the process itself but rather a context-dependent outcome shaped by signal intensity, cellular homeostasis, and interplay with other cell death modalities.

### 3.5. Metabolic Reprogramming Mediates Drug Resistance

#### 3.5.1. Metabolic Reprogramming in Cancer Cells

Metabolic reprogramming encompasses the fundamental, systematic changes in intracellular metabolic pathways that cancer cells undergo to meet growth demands within the hypoxic and nutrient-deprived TME [138]. The core manifestation is the systemic remodeling of the three major intracellular nutrient metabolic pathways [138], the most common of which is aerobic glycolysis, known as the Warburg effect [138]. Enhanced glycolysis in tumor cells is often associated with drug resistance [139].

#### 3.5.2. Multiple Mechanisms by Which Metabolic Reprogramming Mediates PTX Resistance

PTX resistance can be mediated by metabolic reprogramming through many key factors or signaling pathways. Carbonic anhydrase IX (CAIX), a transmembrane metalloproteinase that regulates acid–base balance and metabolic reprogramming in gastric cancer cells, is highly expressed in various cancers, where it serves as a biomarker of poor prognosis and can mediate PTX resistance [140,141,142]. Cancer-associated fibroblasts (CAFs) secrete angiopoietin-like protein 4 (ANGPTL4), which activates the IQGAP1/Raf/MEK/ERK/peroxisome proliferator-activated receptor γ coactivator 1α (PGC1α) signaling axis in prostate cancer cells, inducing mitochondrial biogenesis and metabolic reprogramming toward oxidative phosphorylation, thereby mediating taxane resistance [143]. FoxO6 promotes glycolytic metabolic reprogramming in hepatocellular carcinoma cells by activating the PI3K/Akt signaling pathway, thereby mediating PTX resistance [144]. GLUT1 promotes proliferation, invasion, and glycolysis in endometrial cancer cells while simultaneously conferring PTX resistance [139].

Metabolic reprogramming does not operate in isolation but is deeply intertwined with the other resistance mechanisms discussed above. Enhanced aerobic glycolysis, for example, not only provides rapid ATP for drug efflux pumps such as P-gp but also generates lactate, which acidifies the tumor microenvironment and promotes an immunosuppressive niche that facilitates PTX resistance (see Section 3.8). Moreover, key glycolytic intermediates and metabolites can directly influence apoptotic thresholds by modulating mitochondrial function and ROS production, thereby connecting metabolic rewiring to the dysregulation of the intrinsic apoptosis pathway (see Section 3.3.6). In addition, nutrient-sensing pathways, including the PI3K/Akt/mTOR axis, simultaneously drive both anabolic metabolism and the expression of ABC transporters, illustrating how upstream signaling coordinates metabolic adaptation with drug efflux in resistant cells. Therefore, targeting metabolic vulnerabilities may not only disrupt the energy supply of drug-resistant cells but also simultaneously interfere with multiple downstream resistance pathways, offering a multi-pronged therapeutic strategy.

### 3.6. Epigenetic and Non-Coding RNA Regulation of PTX Resistance

Epigenetic modifications, including DNA methylation, histone acetylation, and non-coding RNA regulation, have been shown to mediate PTX resistance.

#### 3.6.1. Non-Coding RNA Regulation

Non-coding RNAs (ncRNAs) are RNA transcripts that do not encode proteins and are classified into small non-coding RNAs (sncRNAs) and long non-coding RNAs (lncRNAs) [145]. Various microRNAs (miRNAs), lncRNAs, and circular RNAs (circRNAs) play key roles in cancer drug resistance, particularly chemotherapy resistance [145]. Recent studies have revealed that the development of PTX resistance is associated with non-coding RNA regulation.

##### Long Non-Coding RNA (lncRNA)

(1) Overview of lncRNAs and its role in cancer

lncRNAs are a class of non-coding transcripts longer than 200 nucleotides that lack protein-coding potential and participate in the regulation of various cellular processes [145]. lncRNAs are aberrantly expressed in cancer cells and can be categorized into oncogenic lncRNAs and tumor-suppressive lncRNAs, which modulate nearly all malignant biological behaviors of cancer cells via multi-layered regulation. Usually, lncRNAs can widely affect cancer development by regulating cell proliferation and the cell cycle; modulate cell apoptosis and autophagy; promote or suppress invasion, migration, and EMT; sustain the stemness of cancer stem cells; mediate resistance to chemotherapy, targeted therapy, and radiotherapy; control tumor metabolic reprogramming; and engage in immune evasion. Their multi-pronged regulatory mechanisms include serving as miRNA sponges in the ceRNA network, recruiting epigenetic complexes to modulate gene transcription, interacting with RNA-binding proteins, regulating alternative splicing, and mediating intercellular communication through exosomes.

(2) Mechanisms of PTX Resistance Mediated by lncRNAs

Emerging studies indicate that various lncRNAs contribute to chemotherapy resistance by regulating wide and distinct target genes and cellular events [145]. Autophagy activation mediated by DNA damage-induced transcript 4 antisense RNA 1 (DDIT4-AS1) promotes TNBC and PTX resistance [146]. HOX Transcript Antisense RNA (HOTAIR) can enhance tumor cell resistance to PTX [147]. Taurine-upregulated gene 1 (TUG1) promotes autophagy-related PTX resistance by binding to miR-29b-3p in ovarian cancer cells [148]. DC-STAMP domain-containing 1 antisense RNA 1 (DCST1-AS1) promotes transforming growth factor β (TGF-β)-induced EMT via annexin A1 (ANXA1) and enhances PTX resistance in triple-negative breast cancer cells [149]. The long non-coding RNA DANCR (Differentiation Antagonist of Non-coding RNA) acts on miR-33b5p to regulate lactate dehydrogenase (LDHA) expression, thereby enhancing glucose uptake and glycolytic rate, ultimately mediating PTX resistance through metabolic reprogramming [150]. Significant upregulation of small nucleolar RNA host gene 1 (SNHG1) enhances glucose metabolism in the PTX-resistant cell line MKN-45 TXR [151].

##### MicroRNAs (miRNAs)

(1) The Role of miRNAs in Cancer

MicroRNAs (miRNAs) are a class of small non-coding RNAs, typically 19–24 nucleotides in length, that suppress gene expression at the post-transcriptional level by binding to the 3′-untranslated region (3′-UTR) of target messenger RNAs (mRNAs) and other RNA transcripts [145,152,153]. Numerous miRNAs are dysregulated in cancer and play pivotal roles in tumorigenesis, metastasis, and the development of resistance to chemotherapy and radiotherapy [145].

(2) Mechanisms of miRNA-Mediated PTX Resistance

Several oncogenic miRNAs, including miR-21 and miR-155-5p, promote PTX resistance [145,154,155]. Conversely, certain miRNAs can suppress PTX efficacy: miR-140-5p targets E2F transcription factor 3 (E2F3) [156], and downregulation of miR-24-3p mediates resistance in prostate cancer [157]. Downregulation of miR-451a, coupled with upregulation of ADAM10 (a disintegrin and metalloproteinase 10), enhances PTX resistance in hepatocellular carcinoma cells [158]. Furthermore, the miR-155-5p expression level is significantly positively correlated with PTX resistance [159]. Many miRNAs are therefore identified as key players in PTX resistance.

##### Circular RNAs (circRNAs)

(1) Overview of circRNAs and Their Role in Tumorigenesis

Circular RNAs (circRNAs) are a novel class of non-coding RNAs (ncRNAs) that lack 5′ and 3′ ends and possess a distinctive circular structure [145,160,161]. CircRNAs are aberrantly expressed in tumors, where they can be classified into oncogenic circRNAs and tumor-suppressive circRNAs. circRNAs are increasingly recognized as key regulatory factors in the development of various cancers, underscoring their importance in tumorigenesis and drug resistance [160,161]. circRNAs usually act as a “sponge” for microRNAs (miRNAs), influencing the function of miRNA target genes [145,161].

(2) circRNA-Mediated PTX Resistance

Recent studies have indicated that circRNAs play an important role in PTX resistance. Several circRNAs have been implicated in gastric cancer resistance [145]. Both circCELSR1 and circTNPO3 are significantly overexpressed in ovarian cancer and are associated with PTX resistance [162,163]. Circ_0006528 acts as a sponge for miR-1299 to upregulate CDK8 expression, thereby partially contributing to PTX resistance in breast cancer cells [164]. circRNF111 is upregulated in breast cancer tissues and cell lines and mediates PTX resistance [156].

##### Common Molecular Mechanisms Underlying ncRNA-Mediated PTX Resistance

Collectively, these examples illustrate that lncRNAs, miRNAs, and circRNAs often converge on a limited set of core resistance pathways. A frequent mechanism is the competitive endogenous RNA (ceRNA) network, in which lncRNAs and circRNAs act as molecular sponges to sequester miRNAs, thereby relieving the suppression of their downstream targets—such as oncogenes, glycolytic enzymes, or anti-apoptotic factors—that drive PTX resistance [145,146,147,148,149,150,151,152,153,154,155,156,157,158,159,160,161,165]. For instance, many of the ncRNAs identified above ultimately impinge on the PI3K/Akt, MAPK/ERK, or NF-κB signaling cascades, as well as on key phenotypic switches like EMT and metabolic reprogramming, regardless of their initial targets. This convergence suggests that ncRNAs function as regulatory hubs that fine-tune the expression of multiple resistance-associated genes and cellular events simultaneously, rather than acting through isolated targets or linear pathways. Therefore, understanding these shared regulatory molecular networks may reveal common therapeutic vulnerabilities that can be targeted to overcome ncRNA-mediated PTX resistance.

#### 3.6.2. DNA Methylation

(1) Overview of DNA Methylation and Its Role in Regulating Tumor Growth

DNA methylation is an epigenetic mechanism that regulates gene expression and chromatin structure. It influences gene expression in complex ways [166]. Abnormalities in epigenetic regulation within cells can alter the expression of key genes, which may be associated with the initiation, progression, and maintenance of cancer [167].

(2) DNA Methylation-Mediated PTX Resistance

Multiple studies have demonstrated that DNA methylation contributes to PTX resistance in tumor cells. In PTX-resistant MCF-7/Taxol breast cancer cells, the GSDME enhancer region is hypermethylated, leading to the downregulation of GSDME expression. Treatment with the demethylating agent Decitabine reverses this methylation, restores GSDME expression, and induces pyroptosis, thereby enhancing the chemosensitivity of these resistant cells to PTX [45]. DNA methyltransferase 1 (DNMT1) increases methylation at the Krüppel-like factor 4 (KLF4) promoter, thereby downregulating KLF4 expression and mediating PTX resistance in breast cancer cells [168]. Methylation of MEG3 may promote breast cancer cell proliferation and metastasis, thereby enhancing chemoresistance to PTX [169].

#### 3.6.3. Histone Modifications and Chromatin Remodeling

(1) Overview of Histone Modifications and Chromatin Remodeling and Their Role in Regulating Tumor Growth

Reversible covalent modifications on histone tails mainly include acetylation, methylation, ubiquitination, and phosphorylation. Histone acetylation can activate oncogenes, inhibit apoptosis, and accelerate cell cycle progression. Histone methylation drives the transcription of genes associated with cell proliferation and stemness. Histone ubiquitination stabilizes repressive chromatin, facilitating cancer stemness and drug resistance. Abnormal chromatin remodeling results in genomic instability and accelerated accumulation of mutations, as well as disrupted chromatin accessibility and the shutdown of tumor-suppressive pathways. Aberrant recruitment of chromatin remodelers to oncogenic enhancers sustains unlimited proliferation of tumor cells and promotes tumorigenesis.

(2) DNA Histone Modifications and Chromatin Remodeling-Mediated PTX Resistance

Histone acetylation also affects PTX resistance. This modification changes chromatin accessibility and gene expression, which shifts the balance between pro-apoptotic and anti-apoptotic genes toward cancer cell survival [170,171]. Some studies indicate that PTX regulates p-gp expression, which affects PTX levels in cells and causes the development of drug resistance through regulating histone acetylation [69]. Long-term PTX treatment drives adaptive super-enhancer reprogramming via remodeling histone modifications [172]. Super-enhancers sustain persistent and robust transcription of drug resistance genes; consequently, the resistant phenotype can be maintained even after drug withdrawal. Epigenetic plasticity (dynamic chromatin remodeling) enables tumor cells to rapidly adapt to chemotherapeutic pressure without acquiring new DNA mutations. This phenomenon explains why PTX resistance still emerges in many patients in the absence of *TUBB3* mutations.

### 3.7. Chromosomal Instability-Mediated PTX Resistance

(1) Overview of Chromosomal Instability (CIN) and Its Role in Tumors

Chromosomal instability (CIN) refers to the persistent gain and loss of chromosomes or chromosomal segments, leading to changes in chromosome number (i.e., aneuploidy) and structural alterations. CIN is present in most cancers and is associated with poor patient prognosis across various cancer types [173,174,175]. Although CIN drives genomic heterogeneity, it also induces cellular stress and can cause cell cycle arrest. Notably, aneuploidy itself is a common feature of human tumors [176].

(2) Chromosomal Instability Mediates PTX Resistance

Inducing a cycle of chromosomal instability (CIN) in human cells can accelerate the acquisition of resistance to anticancer therapies in cancer cells [176]. G1 phase arrest—a common feature of aneuploidy—renders cancer cells resistant to chemotherapeutic agents [173]. The acquisition of a single chromosome enhances cancer cell resistance to PTX [173]. The transition from abnormal multipolar spindles to normal bipolar spindles significantly reduces chromosomal instability and confers resistance to PTX in cancer cells [177].

### 3.8. TME-Mediated Drug Resistance

(1) Overview of the TME and Its Role in Tumors

The TME is a dynamic and complex ecosystem composed of diverse cell types (including tumor cells, immune cells, and fibroblasts) and non-cellular components (such as the extracellular matrix, growth factors, and cytokines) [178,179,180]. Studies have shown that non-cancerous cells within the TME play a crucial role in tumor initiation and progression by supporting tumor survival, metastasis, and disease progression [181].

(2) The TME Mediates PTX Resistance

The tumor immune microenvironment is closely associated with drug resistance. For instance, hypoxia within the TME induces the upregulation of glycoprotein 96 (gp96) both intracellularly and extracellularly; gp96 subsequently degrades p53, thereby reducing the sensitivity of breast cancer cells to PTX [182]. Moreover, the immunosuppressive remodeling of the TME plays a critical role in PTX resistance. Specifically, an increase in regulatory T cells (Tregs) and the polarization of tumor-associated macrophages toward the immunosuppressive M2 phenotype have been shown to dampen antitumor immunity and facilitate the survival of drug-resistant tumor cells, thereby contributing to PTX resistance [55,58]. In triple-negative breast cancer, functional shifts in cancer-associated fibroblast (CAF) subtypes within the TME are associated with PTX resistance [183]. Overexpression of C-C chemokine ligand 2 (CCL2) activates the PI3K/Akt pathway, thereby promoting macrophage migration and contributing to PTX resistance in human ovarian cancer cells [184]. Furthermore, aberrant angiogenesis within the TME contributes significantly to PTX resistance; PTX can activate Toll-like receptor 4 (TLR4) on tumor cells, triggering systemic inflammatory responses that promote both angiogenesis and de novo lymphangiogenesis within the TME, thereby facilitating tumor metastasis and further compromising therapeutic efficacy [57]. Additionally, intercellular communication via small extracellular vesicles (sEVs) represents an important mode of resistance transfer within the TME. Drug-resistant tumor cells can package miRNAs, such as miR-155-5p, into sEVs and deliver them to neighboring sensitive cells, thereby disseminating chemoresistance and promoting EMT in recipient cells [155,158]. The TME and its effects are very complicated and include hypoxia, inflammatory and suppressive immune cell phenotype transition, CAF subtype functional shift, and intercellular communication via small extracellular vesicles, which can contribute to PTX resistance.

### 3.9. Cell Plasticity-Mediated PTX Resistance

(1) Overview of the Cell Plasticity and Its Role in Tumors

Along with the metabolic reprogramming and immune adaption described in the sections above, other types of cancer cell plasticity include epithelial–mesenchymal plasticity (EMT), cancer stem cell (CSC) plasticity, lineage plasticity, and polyploid giant cancer cell plasticity. Of these, epithelial–mesenchymal plasticity and CSC plasticity have been well investigated. EMT is not a binary switch but a dynamic and reversible process that enables carcinoma cells to adopt hybrid epithelial/mesenchymal states. These intermediate states are associated with enhanced tumor-initiating capacity and drug tolerance and may coexist with the CSC phenotype. Cells with mesenchymal features and stemness properties are more prone to enter a quiescent or slow-cycling state and become drug-tolerant persister cells (DTPs).

(2) Cell Plasticity-Mediated PTX Resistance

Cancer cells undergo reversible cell state transitions independent of DNA sequence mutations via epigenetic reprogramming and transcriptional network remodeling, which constitutes the core basis of non-genetic drug resistance, metastasis, and tumor recurrence. Epithelial–mesenchymal plasticity enables cancer cells to lose epithelial junctions, acquire migratory and invasive capacities, upregulate stemness features, facilitate DTP formation, and confer tolerance to PTX and other chemotherapeutic agents [185,186]. Under chemotherapeutic stress, differentiated cells can be reprogrammed to gain stem cell properties. CSCs generally exhibit slow proliferation and enhanced DNA damage repair capacity, rendering them resistant to PTX [187]. DTPs display reduced sensitivity to anti-mitotic drugs such as PTX (a microtubule poison); this is transient drug tolerance rather than permanent resistance [188]. Some studies have demonstrated that PTX can induce epithelial–mesenchymal transition and the generation of CSCs.

### 3.10. Summary of PTX Resistance

As summarized above, PTX resistance is very complicated and can be divided into two types: intrinsic and/or acquired resistance. There are shared mechanisms of resistance common to most cancer types, e.g., alterations in tubulin targets, drug efflux, dysregulation of classical proliferation and apoptosis signaling pathways, excessive autophagy activation, changes in cell plasticity, e.g., EMT and cancer cell stemness, epigenetic modifications, metabolic reprogramming, and tumor microenvironment remodeling.

As well as these shared resistance mechanisms, different cancer types have specific personalized molecular or cellular mechanisms (see Table 1), which indicates that the problem of PTX resistance might involve customized therapy.

Several conceptual distinctions should be emphasized when interpreting the resistance mechanisms discussed above. First, adaptive drug tolerance—in which cancer cells enter a reversible, slow-cycling persister state that transiently reduces sensitivity to PTX—should be differentiated from stable acquired resistance driven by genetic mutations or persistent epigenetic alterations. Drug-tolerant persister cells (DTPs) have been observed under PTX treatment (Section 3.9), and their transient, reversible nature implies distinct therapeutic strategies compared with irreversible resistance mechanisms. Second, although this review focuses specifically on PTX, several of the mechanisms described—such as upregulation of P-gp (Section 3.2) or alterations in microtubule dynamics (Section 3.1)—may also confer cross-resistance to other taxanes (e.g., docetaxel) or even to structurally unrelated microtubule-targeting agents. Third, many of the molecular alterations summarized in this review represent mechanistic associations with the resistant phenotype; a causal role has been functionally validated through gain- or loss-of-function experiments only for a subset. Finally, most of the molecules and gene signatures discussed remain at the stage of proposed biomarkers; very few have undergone rigorous prospective clinical validation for guiding PTX-based therapy. These distinctions are essential for prioritizing therapeutic targets and for designing clinically informative biomarker studies.

## 4. Reversal of PTX Resistance and Clinical Translation Strategies

As evident from the preceding discussion, the mechanisms underlying PTX resistance are highly complex. Some strategies aimed at reversing resistance by targeting core mechanisms are summarized below.

### 4.1. Development of New Drugs Targeting Microtubule-Associated Proteins (MAPs)

Overexpression of TUBB3 (βIII-tubulin), point mutations in the coding region of β-tubulin, remodeling of microtubule-associated proteins (MAPs), and dysregulated post-translational modifications of tubulin mediate PTX resistance. Accordingly, novel tubulin-regulating agents can be developed. One strategy is to select microtubule stabilizers that do not rely on wild-type βI-tubulin yet retain binding affinity toward βIII-tubulin. For instance, epothilones remain active against PTX-resistant tumors with high TUBB3 expression and have been approved for metastatic breast cancer resistant to taxanes [199,200]. Alternative approaches include small-molecule and nucleic acid therapeutics that inhibit or downregulate TUBB3 (βIII-tubulin) expression. In addition, stathmin inhibition can attenuate microtubule depolymerization, facilitate microtubule acetylation, and modulate microtubule dynamics as well as tubulin post-translational modifications. Novel combinatorial regimens can also be adopted to block downstream resistance pathways mediated by TUBB3.

### 4.2. Development of Inhibitors Targeting P-gp and Other Efflux Pumps

Current evidence indicates that overexpression of the P-gp efflux pump in cancer cells is a major driver of MDR, and the development of P-gp inhibitors represents a rational strategy to reverse PTX resistance [67]. P-gp inhibitors have been developed for several generations, but none have been approved for use. These include (1) verapamil and cyclosporine A; (2) Valspodar (PSC-833), Biricodar (VX-710), and Dexverapamil; and (3) Tariquidar (XR9576) and Zosuquidar (LY335979). These P-gp inhibitors are still in the stage of research and development. Several agents have shown promise in this regard. Polyphyllin H reverses PTX resistance by targeting cholesterol rafts to inhibit multiple ABC transporters [68]. A non-humanized chimeric bispecific antibody targeting P-glycoprotein and CD47 has been demonstrated to effectively restore tumor sensitivity to PTX [67]. Apigenin enhances cellular sensitivity to PTX by downregulating ABCB1/MDR1 expression and consequently inhibiting membrane transport function [66]. The dual-target PI3K/mTOR inhibitor DHW-221 functions as a P-gp inhibitor by binding to P-gp, thereby reducing its expression and activity, and holds promise for overcoming PTX resistance [73].

### 4.3. Development of Inhibitors Targeting Regulatory Signaling Pathways

Multiple signaling pathways, including PI3K/Akt, MAPK, and NF-κB, are involved in PTX-induced tumor resistance. The research and development of inhibitors targeting these pathways can therefore enhance tumor sensitivity to PTX.

(1) Modulation of the PI3K/Akt signaling pathway

PI3K/Akt is a very important signaling pathway responsible for cancer cell growth and proliferation and is associated with PTX resistance. Cajanol is an isoflavone isolated from pigeon pea (Cajanus cajan); it downregulates the expression of P-gp via the PI3K/Akt/NF-κB pathway, thereby inhibiting PTX efflux in A2780/PTX cells and reversing their resistance to PTX [77]. Radix Tetrastigma Extracts (RTEs) enhance the chemoresponsiveness of PTX-resistant triple-negative breast cancer cells by inhibiting PI3K/Akt/mTOR-mediated autophagy [201].

(2) Modulation of the MAPK/EKR signaling pathway

The MAPK/ERK pathway can regulate P-gp-mediated PTX resistance. The MEK inhibitor U0126 enhances the efficacy of PTX against ovarian cancer cells by inhibiting both the basal and chemotherapy-induced phosphorylation of ERK, as well as MAP kinase-interacting kinase 1 (Mnk1) and eukaryotic translation initiation factor 4E (eIF4E) [82]. Ganoderma lucidum extract may inhibit P-glycoprotein-mediated drug efflux via the MAPK pathway, thereby effectively overcoming PTX resistance in breast cancer [86]. In cervical cancer cells, PTX activates ERK, whereas cobimetinib reverses this activation; consequently, cell growth is inhibited, and caspase-dependent apoptosis is induced through suppression of the MAPK/ERK pathway [88].

(3) Development of strategies targeting the NF-κB signaling pathway

The NF-κB signaling pathway is very important for cancer cell proliferation and survival, and its activation is associated with PTX resistance. The proteasome inhibitor N-acetyl-leucyl-leucyl-norleucyl (ALLN) can inhibit PTX-induced NF-κB activation and restore cellular sensitivity to PTX [95]. Co-treatment with the bisindole alkaloid voacamine (VOA) and PTX inhibits the nuclear translocation of the transcription factor NF-κB, suppresses the growth of ovarian drug-resistant cancer cells, and induces their apoptosis [202].

### 4.4. Modulation of AURKA Levels

AURKA is a protein that is important in mediating PTX resistance. Phenethyl isothiocyanate (PEITC) targets Aurora kinase A to reverse chemotherapy resistance and restore sensitivity to PTX [203]. The novel AK-A inhibitor CYC3, in combination with PTX, exerts the synergistic inhibition of pancreatic cancer cell growth and induces mitotic arrest, potentially overcoming tumor resistance to PTX [106].

### 4.5. Modulation of FOXM1 Levels

FOXM1 also is a key factor to regulate cell survival and involve PTX resistance. The class I histone deacetylase (HDAC) inhibitor domatinostat suppresses FOXM1 expression and function, thereby sensitizing pancreatic cancer to first-line standard gemcitabine/PTX dual-agent chemotherapy [204]. Concurrently, domatinostat effectively targets the FOXM1–Survivin axis that is essential for ovarian cancer cell viability; this inhibitory effect is further enhanced when combined with PTX [205].

### 4.6. Modulation of BCL-2 Family Levels

BCL-2 is a family of anti-apoptosis proteins that attenuate the cytotoxic effect of PTX. The Bcl-2 inhibitor venetoclax enhances the efficacy of albumin-bound PTX (nab-PTX) in patient-derived xenograft (PDX) models of Bcl-2-overexpressing, treatment-resistant Ewing sarcoma [206]. Lisaftoclax (APG-2575) and Sonrotoclax (BGB-11417) were shown to work by targeting BCL-2 or BCL-2 mutations and were also approved; however, it remains unclear whether they can improve PTX resistance. Some drug combinations can indirectly regulate BCL-2. Combination therapy with Duloxetine and PTX promotes the mitochondrial localization of activated cyclin-dependent kinase 1 (CDK1), leading to phosphorylation of the anti-apoptotic proteins Bcl-2 and Bcl-xL and thereby triggering apoptotic cell death in PTX-resistant ovarian cancer cells [207]. Nanovesicles co-loaded with PTX and doxorubicin downregulate BCL-2 and upregulate Bax, thereby inducing apoptosis and overcoming PTX resistance in gastric cancer cells [208].

### 4.7. Targeting GR Signaling to Enhance Taxane Efficacy

GR antagonism represents an emerging strategy to enhance taxane efficacy in treatment-resistant settings. Relacorilant, a selective GR antagonist (SGRA), specifically blocks GR signaling without interfering with progesterone receptors or other steroid receptors, thereby minimizing off-target effects [119]. Preclinical studies have demonstrated that GR blockade restores sensitivity to taxane-based therapies by reversing GR-mediated suppression of apoptosis [124]. In OVCAR5 cells in vitro and in the MIA PaCa-2 xenograft, the glucocorticoid receptor (GR) modulator relacorilant with nab-paclitaxel induced significant tumor regression, providing a mechanistic rationale for clinical translation [209].

The ROSELLA trial was a pivotal, global, randomized, open-label, phase 3 study designed to evaluate the efficacy of relacorilant, a first-in-class SGRA, combined with nab-paclitaxel in PROC [210]. It should be noted that this trial enrolled patients with platinum-resistant rather than PTX-resistant disease. The preliminary results from the ROSELLA trial establish that the combination of relacorilant and nab-paclitaxel targeting GR signaling can enhance chemotherapy efficacy in patients, translating a biological concept into clinical benefit, although its role in directly reversing established PTX resistance requires further investigation [120].

### 4.8. Regulation of Autophagy

Protective autophagy can enhance PTX resistance. It is necessary to inhibit this protective autophagy via related key proteins or signaling pathways. The PI3K/AKT inhibitors ipatasertib and taselisib can induce enhanced autophagy signaling in PI3K/AKT-resistant triple-negative breast cancer cells and exhibit synergistic effects with taxane-based chemotherapy [136]. By inhibiting autophagy, chloroquine prevents A549 cells from developing PTX resistance over time and enhances the effects of PTX by increasing the accumulation of damaged mitochondria that produce superoxide radicals [211].

### 4.9. Epigenetic Regulation

Epigenetic modifications can widely affect PTX resistance in cancer cells. It is useful to develop epigenetic modified drugs to enhance PTX sensitivity. 3,3′-Diindolylmethane (DIM) enhances the antitumor effects of PTX on MCF-7 and T47D cells by regulating the expression of DNA methyltransferase 1 (DNMT1) and Krüppel-like factor 4 (KLF4) [168]. Decitabine (5-Aza-2′-deoxycytidine) enhances GSDME expression and induces pyroptosis through DNA demethylation, thereby increasing the chemoresponsiveness of MCF-7/PTX cells to PTX [45].

### 4.10. Regulation of Chromosomal Instability

Chromosomal instability can cause PTX resistance, and corresponding tactics can target chromosomal instability. The microtubule inhibitor S-72 blocks the activation of the stimulator of interferon genes (STING) in PTX-resistant breast cancer cells, thereby restoring the formation of multipolar spindles, leading to lethal chromosomal instability and consequently overcoming PTX resistance [212]. However, the anticancer effect of S-72 seems to be not directly related to regulating chromosomal instability due to its activity on the colchicine binding site. Inhibition of salt-induced kinase 2 (SIK2) by the novel selective inhibitor MRIA9 increases chromosomal instability, thereby enhancing the sensitivity of ovarian cancer cell lines and patient-derived 3D spheroids to PTX [213].

### 4.11. Regulation of Metabolic Reprogramming

Since metabolic reprogramming is often seen in cancer cells, interferences in cancer cell metabolism may be able to increase the sensitivity to PTX treatment. Treatment with hexokinase 2 (HK2) inhibitors restores PTX sensitivity, cell viability, and oxygen consumption rates following PTX treatment; additionally, downstream expression levels of ABC and solute carrier (SLC) transporters are reduced in ovarian clear cell carcinoma (OCCC) cells [214]. FV-429, a derivative of the natural flavonoid wogonin, reprograms the metabolic processes of cancer cells, reduces fatty acid levels, and thereby increases the sensitivity of these cancer cells to PTX [215].

### 4.12. Modulation of the TME

Targeting the TME is an emerging approach to overcoming PTX resistance, with strategies including reversing hypoxia, remodeling the extracellular matrix, or reprogramming immunosuppressive cells. For example, in patients with TNBC, a PD-L1 antibody atezolizumab in combination with nab-paclitaxel-based chemotherapy significantly improved pathological profiles and showed acceptable safety to these patients (Phase 3 trial) [216]. Many basic studies also support the tactics of targeting the TME. Alpha-hederin sensitizes PTX-resistant non-small-cell lung cancer (NSCLC) cells by mediating the accumulation of various microRNAs (miRNAs) through small extracellular vesicles (sEVs) and by inhibiting the transforming growth factor β/SMAD2 signaling pathway in recipient cells [217]. Furthermore, nanotechnology-based TME modulation strategies have shown promise. Specifically, PTX-loaded ginsenoside Rg3 liposomes (Rg3-PTX-LPs) can simultaneously target MCF7 cancer cells and remodel the TME by repolarizing tumor-associated macrophages from the immunosuppressive M2 phenotype to the antitumor M1 phenotype, suppressing myeloid-derived suppressor cells (MDSCs), and decreasing tumor-associated fibroblasts (TAFs) and collagen fibers through inhibition of the IL-6/STAT3/p-STAT3 pathway, thereby significantly reversing PTX resistance and enhancing antitumor efficacy in vivo [218].

As evident from the above, a multitude of strategies have been developed for reversing PTX resistance. These strategies are summarized in Table 2.

### 4.13. Comparative Assessment of Current Strategies for Overcoming PTX Resistance

The strategies summarized in Table 1 can be critically compared along several dimensions based on the evidence presented in the literature. First, these strategies differ substantially in their molecular targets and mechanisms of action. Some approaches directly target individual resistance effectors: for example, P-gp inhibitors such as Polyphyllin H and Apigenin block drug efflux [66,68], while Bcl-2 inhibitors such as Venetoclax directly promote apoptosis in resistant cells [206]. In contrast, strategies targeting upstream signaling pathways—such as inhibitors of PI3K/Akt (Cajanol, RTEs) [77,201], MAPK/MEK (U0126, Cobimetinib) [82,88], and NF-κB (ALLN, VOA) [95,202]—may simultaneously modulate multiple downstream resistance mechanisms. RTEs, for instance, inhibit PI3K/Akt/mTOR-mediated autophagy in addition to suppressing survival signaling [201]. Similarly, DHW-221 functions as both a dual PI3K/mTOR inhibitor and a P-gp inhibitor, representing a strategy that concurrently targets signal transduction and drug efflux [73].

Second, the chemical nature and delivery modality of these strategies present distinct profiles. As shown in Table 1, a substantial proportion of the reversal agents are natural products or their derivatives, including Polyphyllin H [68], Cajanol [77], RTEs [201], Ganoderma lucidum extract [86], PEITC [203], VOA [202], DIM [168], FV-429 [215], and Alpha-hederin [217]. Others are synthetic small-molecule inhibitors (e.g., DHW-221 [73], U0126 [82], Cobimetinib [88], CYC3 [106], Domatinostat [204,205], Venetoclax [206], and MRIA9 [213]). A third category includes macromolecular or nanoformulation-based approaches, such as the non-humanized chimeric bispecific antibody targeting P-gp and CD47 [67], nanovesicles co-loaded with PTX and doxorubicin [208], and PTX-loaded ginsenoside Rg3 liposomes (Rg3-PTX-LPs) [218]. The latter category is designed to enable the simultaneous delivery of multiple agents or to achieve the dual targeting of tumor cells and the tumor microenvironment. For example, Rg3-PTX-LPs target both MCF7 cancer cells and the TME by repolarizing tumor-associated macrophages from the M2 to M1 phenotype, suppressing MDSCs, and decreasing TAFs and collagen fibers through the IL-6/STAT3/p-STAT3 pathway [218].

Third, the cancer types and experimental models in which these strategies have been tested vary considerably. Certain combination regimens have been evaluated in specific cancer contexts: RTEs with PTX in triple-negative breast cancer [201], Cobimetinib with PTX in cervical cancer [88], CYC3 with PTX in pancreatic cancer [106], VOA with PTX in ovarian cancer [202], Venetoclax with nab-PTX in Ewing sarcoma PDX models [206], MRIA9 with PTX in ovarian cancer cell lines and patient-derived 3D spheroids [213], HK2 inhibitor with PTX in ovarian clear cell carcinoma [214], and Alpha-hederin with PTX in non-small-cell lung cancer [217]. The diversity of cancer types tested underscores the broad applicability of these strategies yet also highlights that the efficacy of each approach may be context-dependent.

Fourth, although epothilones have been clinically approved to be effective in PTX-resistant breast cancer, this drug still has severe side effects, e.g., neuron toxicity. For p-gp inhibitors, some drugs were claimed to increase sensitivity to PTX treatment, but they usually have off-target pharmacological activities (e.g., verapamil affects the heart) or toxicities (e.g., Valspodar strongly inhibits CYP3A4). Some human trials have been conducted, but no studies indicate that cancer patients can benefit from this combined therapy. Several agents that are clinically approved for other indications have shown preclinical potential to enhance PTX sensitivity, including Cobimetinib (approved for BRAF-mutant melanoma), Venetoclax (approved for CLL/AML), and Decitabine (approved for MDS/AML). However, their combination with PTX for reversing PTX resistance remains at the preclinical stage, and their approved indications do not constitute clinical validation of these specific PTX-based combinations. Similarly, non-anticancer drugs such as Duloxetine and chloroquine have demonstrated PTX-sensitizing effects in preclinical models, but evidence for their clinical efficacy in this context is lacking. Domatinostat, Ipatasertib, and PEITC are in the stage of phase I or II clinical trials. Among emerging strategies, relacorilant, a selective GR antagonist, has completed a phase III trial (ROSELLA), demonstrating significant overall survival benefit in combination with nab-paclitaxel in platinum-resistant ovarian cancer. However, it should be noted that this represents enhancement of taxane efficacy in a platinum-resistant rather than a PTX-resistant population, and its role in directly reversing established PTX resistance requires further investigation. In summary, while several strategies have reached clinical evaluation in related treatment-resistant settings—notably, the phase III ROSELLA trial of relacorilant plus nab-paclitaxel in platinum-resistant ovarian cancer—no agent has yet received regulatory approval specifically for reversing established PTX resistance in PTX-resistant tumors. It should be noted that relacorilant plus nab-paclitaxel received regulatory approval in March 2026 for enhancing taxane efficacy in platinum-resistant ovarian cancer, which is distinct from an approval for reversing PTX resistance per se. The majority of candidate reversal strategies remain at the preclinical stage, and a substantial translational gap persists between promising preclinical findings and validated clinical benefit.

In summary, the current landscape of PTX resistance reversal strategies is characterized by mechanistic diversity, a spectrum of chemical modalities from natural products to nanoformulations, and cancer-type-specific preclinical validation. Given that no single strategy has been universally effective, the selection of an appropriate reversal approach should ideally be guided by the dominant resistance mechanism present in a given tumor. This further emphasizes the need to match specific resistance drivers with the most relevant targeted intervention. In addition, we have no ideal specific approved drugs to overcome PTX resistance and need further study.

## 5. Current Challenges and Future Prospects

### 5.1. Current Challenges

Although significant progress has been made in elucidating the mechanisms of PTX resistance, substantial challenges remain in translating these basic research findings into clinical benefits. At the core of these challenges lies the systemic complexity of tumor resistance networks.

(1) Heterogeneity of Cancer Resistance Mechanisms

The spatiotemporal heterogeneity of tumors represents the primary obstacle to overcoming PTX resistance. This heterogeneity manifests at multiple levels. First, the dominant resistance mechanisms vary considerably across cancer types and even among molecular subtypes of the same cancer. For example, triple-negative breast cancer may depend more on metabolic reprogramming and TME remodeling, whereas microtubule alterations and drug efflux pump activity are more prominent in ovarian cancer. Second, intratumoral heterogeneity is high; multiple subclones harboring distinct resistance mechanisms can coexist within a single tumor. The selective pressure exerted by PTX acts as an evolutionary bottleneck, whereby the rare drug-resistant clones present before treatment rapidly expand upon drug exposure, leading to therapeutic failure. Further complicating this scenario, a patient’s resistance phenotype can evolve dynamically over time, with the dominant resistance mechanism potentially shifting during different phases of chemotherapy. This places extremely high demands on serial biopsies and real-time monitoring.

(2) The Complexity of Cross-Regulation Among Multiple Pathways

PTX resistance is driven not by a single linear pathway but by a complex, redundant, and interwoven network of multiple pathways and nodes. A single upstream signaling aberration can simultaneously activate multiple downstream resistance pathways. For example, excessive activation of the PI3K/Akt pathway not only phosphorylates and inactivates the pro-apoptotic protein Bad to inhibit apoptosis (Section 3.3.1) but also enhances NF-κB transcriptional activity, thereby upregulating P-gp expression and drug efflux (Section 3.2.2); concurrently, it can induce protective autophagy (Section 3.4.2), providing a survival refuge for cancer cells. This “one cause, multiple effects” regulatory model suggests that blocking a single node within this network often allows for rapid compensation by unaffected pathways, ultimately leading to therapeutic failure. Moreover, extensive crosstalk between novel mechanisms—such as non-coding RNAs and epigenetic modifications—and classical signaling pathways further deepens the network’s complexity.

(3) Bottlenecks in Translating Preclinical Research to Clinical Practice

A substantial translational gap persists between drug resistance reversal strategies that are effective in vitro and in animal models and their successful implementation in clinical practice. First, conventional two-dimensional (2D) cell lines cultured on plastic surfaces lack the three-dimensional architecture of in vivo tumors as well as relevant cell–cell and cell–matrix interactions. They fail to recapitulate the physiological and pathological conditions of the TME, including hypoxia, acidity, and nutrient deprivation, thereby severely compromising the fidelity of drug response assessments. Second, although patient-derived xenograft (PDX) models and organoids preserve primary tumor characteristics at the histological and genomic levels more faithfully, they still cannot fully replicate the complex immune system, tumor–host interactions, and drug pharmacokinetics inherent to the human body. Finally, drug resistance reversers frequently encounter safety and tolerability challenges in clinical trials. For example, many of the signaling pathways or epigenetic regulators targeted are also essential for normal cellular physiology; systemic inhibition may therefore cause unacceptable toxicity. The most urgent challenge is to achieve precise delivery of reversal agents to tumor tissues, enabling effective antagonism of drug-resistant proteins or pathways while sparing normal physiological functions.

### 5.2. Future Research Directions

To address the challenges outlined above, future research must move beyond traditional single-gene, single-pathway paradigms and adopt systematic, multidimensional integration strategies to propel the study of PTX resistance toward precision and intelligence.

(1) Multi-omics Technologies for Deconstructing Resistance Heterogeneity

Multi-omics technologies, exemplified by single-cell sequencing and spatial transcriptomics, are providing powerful tools to dissect the heterogeneity of drug resistance. By simultaneously profiling the genome, transcriptome, epigenome, or proteome at single-cell resolution, these approaches allow researchers to delineate the resistance landscapes of distinct tumor subclones, identify rare drug-tolerant cells, and trace their dynamic evolutionary trajectories under therapeutic pressure. Spatial transcriptomics, in turn, superimposes gene expression information onto the native architecture of tumor tissue, revealing the spatial organization of different resistant clones in relation to immune cells and fibroblasts within the TME. This makes it possible to unravel the niche-specific mechanisms that foster drug resistance in particular regions. In the future, integrated multi-omics analysis incorporating multi-site and longitudinal sampling will enable the construction of molecular models capable of predicting the emergence of resistance. Such an advance would facilitate a paradigm shift from passive response to proactive early warning of drug resistance and provide precise guidance for the rational design of initial combination regimens.

(2) Optimization of Multi-Target Combination Regimens

Given the complexity of the PTX resistance network, multi-target, multi-pathway synergistic interventions are emerging as an essential strategic direction. Key approaches encompass rationally designed multi-target combinations, immunotherapy-based and TME regulators combinations, as well as the exploration of novel resistance targets—such as chromosomal instability, spindle dynamics, and epigenetic modification—to further identify actionable nodes and enable synergistic targeting. The major strategies are outlined below.

(a) Rational Multi-Target Combination Therapy

More and more protein or kinase targets are emerging in cancer therapy. Rather than focusing on a single resistance mechanism, “cocktail”-style combination regimens can be tailored to the dominant resistance drivers in individual patients. For instance, co-targeting the PI3K/Akt pathway to suppress pro-survival signals, employing Bcl-2 inhibitors to promote apoptosis, and concomitantly blocking drug efflux with P-gp inhibitors can disrupt the resistance network at multiple levels. Identifying and using new “master regulators” capable of coordinately modulating multiple drug resistance-associated targets—such as FOXM1—represents an emerging avenue for the design of highly effective yet low-toxicity combination regimens. In the future, we could use these classic or new regulators on protein or kinase targets to form multi-target combination therapies and enhance their sensitivity to the anticancer effects of PTX.

(b) Combination with Immunotherapy

The immune system plays an important role in cancer prevention and treatment. PTX itself exerts immunomodulatory effects. However, drug-resistant tumors frequently exhibit immune evasion. Combining PTX with immune checkpoint inhibitors (e.g., anti-PD-1/PD-L1 antibodies) or other immunotherapeutic agents holds promise to convert “cold” tumors into “hot” tumors. This phenotypic shift can reactivate immune surveillance to eliminate drug-resistant tumor cells and elicit synergistic, durable antitumor responses. Currently, the majority of clinically available immune checkpoint inhibitors primarily target T lymphocytes. Future efforts should focus on identifying additional immune checkpoint molecules and developing novel immune cell-based therapies targeting cell populations such as Tregs, NK cells, and tumor-associated macrophages. Such strategies may improve tumor sensitivity to PTX-mediated anticancer effects.

(c) Combination with Agents Targeting TME

Along with the immune microenvironment discussed in Section 3.8, aberrant tumor vasculature, a hypoxic microenvironment, and PTX-induced TLR4-driven inflammatory lymphangiogenesis are key drivers of drug resistance and metastasis. Rational sequential administration of anti-angiogenic agents can transiently “normalize” tumor vasculature, improving PTX perfusion and intratumoral distribution while simultaneously alleviating hypoxic stress and attenuating downstream resistance signals. Furthermore, targeting the TLR4 pathway represents a complementary strategy to quell the local and systemic inflammatory circuits that promote malignant progression. In the future, it will be possible to combine these regulators targeting TME to reduce resistance to PTX.

(d) Exploration of Novel Targets on chromosomal instability and epigenetic modification

Along with protein-based anticancer drugs, genetic regulation is emerging as an important tactic for cancer therapy. For example, strategies that deliberately exacerbate chromosomal instability (CIN)—such as SIK2 inhibition or STING pathway disruption—and those that force multipolar spindle-driven mitotic catastrophe (e.g., via HSET/KIFC1 inhibition) represent emerging paradigms for eliminating PTX-resistant tumors. In parallel, targeting the epigenetic plasticity that sustains drug-tolerant states, for instance, by reversing aberrant DNA methylation or disrupting chromatin remodeling complexes, may open complementary avenues to reprogram resistant cells and prevent adaptive rewiring under therapeutic pressure. In the future, we also could combine these genetic or epigenetic regulators with PTX to enhance overall anticancer effects.

(3) Interdisciplinary Integration

Addressing the challenge of drug resistance demands deep interdisciplinary integration across the life sciences, chemistry, materials science, engineering, and computer science. Recently, rapid advances in artificial intelligence (AI), nanotechnology, and organoid technology now offer unprecedented opportunities to revolutionize traditional research paradigms. Harnessing these technologies in a coordinated manner will be critical for advancing PTX resistance research. Key directions are outlined below.

(a) Application of AI in Efficacy Prediction

AI and deep learning algorithms can integrate multi-omics data, pathological images, and clinical information to construct comprehensive predictive models capable of forecasting PTX response and the risk of resistance. These models discern complex patterns in massive datasets that are imperceptible to human analysis. For instance, AI can analyze genomic, proteomic, metabolomic, and single-cell sequencing data to identify resistance drivers; directly infer genetic mutation status and resistance potential from H&E-stained histological sections combined with spatial transcriptomics; and leverage multimodal fusion with large language models to enhance predictive accuracy. Such approaches enable the pre-treatment identification of patients unlikely to benefit from PTX, thereby avoiding ineffective chemotherapy and treatment delays, and represent a core driver of precision prediction.

(b) Pharmaceutical optimization for Overcoming Resistance

PTX formulation, drug exposure, dose schedule, and differences between solvent-based PTX and nab-paclitaxel may influence resistance, and so pharmaceutical optimization, including the differences in PTX formulation, drug exposure, and dose schedule, is necessary. However, in this review, we did not conduct a comparative investigation. In the future, we will conduct comparative studies to clearly indicate that PTX resistance is associated with pharmaceutical formulation and dose schedule.

Nanotechnology offers a versatile platform for circumventing drug efflux and enabling tumor-targeted delivery. Smart nanocarriers can co-encapsulate PTX with resistance modulators (e.g., siRNA or small-molecule inhibitors), exploiting the enhanced permeability and retention (EPR) effect or active targeting via ligand-mediated accumulation to minimize drug efflux. These systems are designed for stimuli-responsive drug release triggered by the TME (e.g., low pH, enzymes, or redox conditions), specifically elevating intratumoral drug concentrations and thereby suppressing the emergence of resistance. Functioning as “Trojan horses,” such nanotherapeutics bypass P-gp efflux pumps to deliver their payload directly into the cytoplasm of resistant cells. By simultaneously reducing systemic toxicity, nanotechnology-based delivery represents one of the most promising avenues in translational research.

(c) Organoid-Based Drug Screening Platforms

Tumor organoids (patient-derived organoids, PDOs) are three-dimensional cultures that can retain selected histological and molecular characteristics of the original tumor. Compared with conventional two-dimensional cell lines, they better recapitulate in vivo drug responses; relative to patient-derived xenograft (PDX) models, they offer shorter turnaround times and higher throughput. For PTX resistance research, paired organoid libraries derived from pre-treatment and post-resistance specimens can be established to dynamically track clonal evolution. Such libraries enable the matrix screening of PTX combined with resistance modulators to optimize therapeutic regimens, forming the basis of an organoid-based drug sensitivity testing platform. Furthermore, organoids have the potential to function as promising ex vivo models and a preclinical platform for the prediction of pre-treatment chemosensitivity, although their ability to predict individual clinical responses requires prospective validation. Current challenges include lack of culture standardization, low establishment rates, high costs, limited throughput, the absence of an immune microenvironment, the inability to recapitulate in vivo drug metabolism, and insufficient toxicological evaluation. In the future, the integration of organoid technology with microfluidic chips and immune cell co-culture systems is anticipated to establish more comprehensive resistance models, enabling low-cost, high-throughput, and high-content drug screening. Beyond pharmacodynamic assessments, the development of organoid systems that emulate in vivo pharmacokinetics and toxicology will accelerate the clinical translation of preclinical findings.

**Figure 4:**
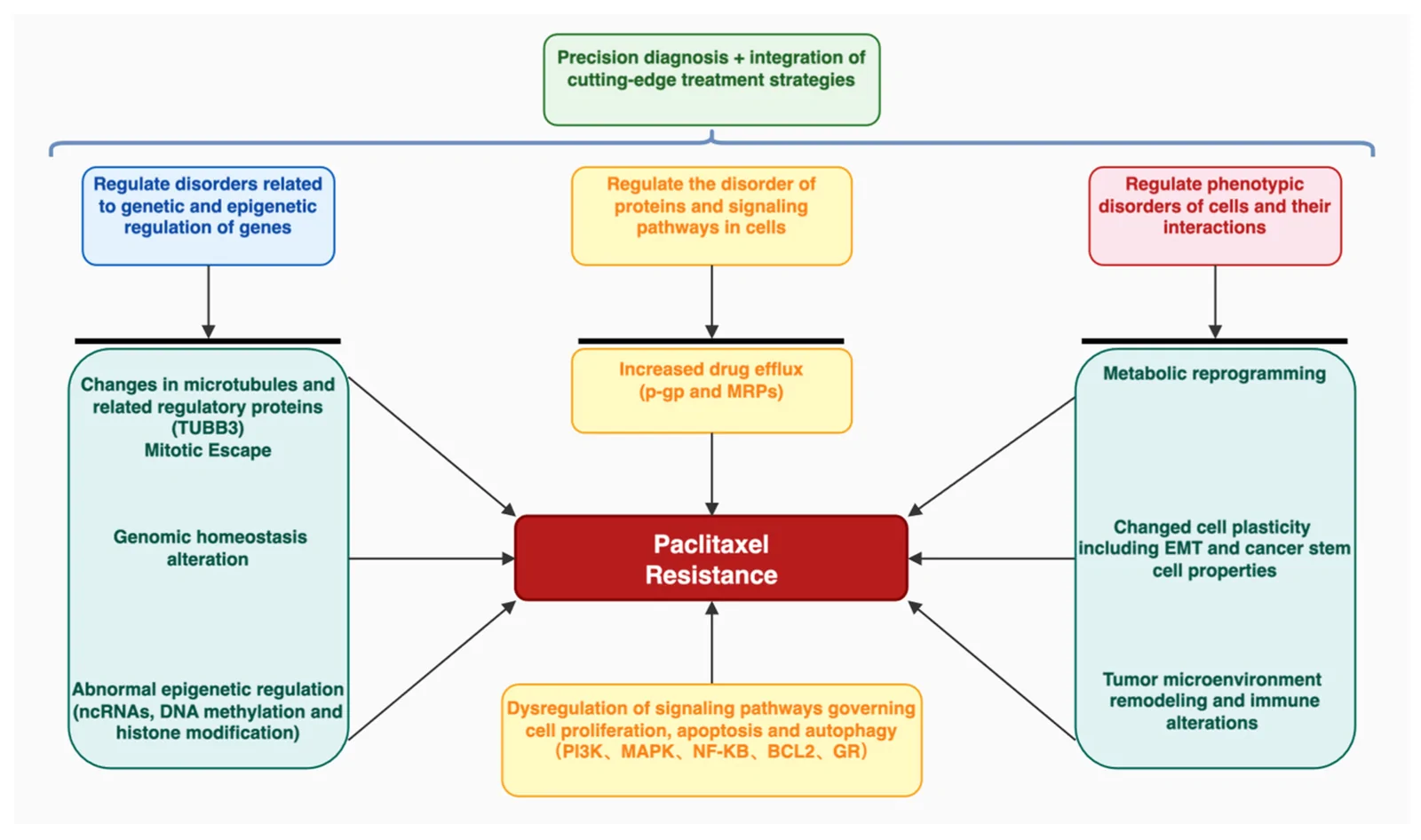
Integrated strategies to regulate molecular and cellular disorders involving PTX resistance.

In summary, PTX is a core first-line chemotherapeutic agent for a broad spectrum of cancers. However, the emergence of intrinsic and acquired resistance critically contributes to chemotherapy failure. PTX resistance is a complex biological process driven by multiple key factors and cross-regulated signaling pathways. It encompasses classical mechanisms, including aberrations in microtubules and related proteins, changes to mitotic systems and chromosomal instability (CIN), epigenetic modifications including ncRNA regulation, DNA methylation and histone modification, enhanced drug efflux, and dysregulation of proliferation and apoptotic signaling pathways, enhanced autophagy, metabolic reprogramming, cell plasticity (EMT and cancer cell stemness), TME remodeling, and immune regulation (see Figure 4). These mechanisms could be grouped into three classical molecular or cellular dysfunctions: genetical and epigenetic disorders, intracellular key proteins and signaling pathway disorders, and cell phenotypic and cell–cell interaction disorders.

Among the diverse mechanisms reviewed, alterations in PTX targets and microtubule dynamics, overexpression of ABCB1/P-gp drug efflux pumps, dysregulation of proliferation and apoptosis through the PI3K/Akt and MAPK/ERK pathways and key factors (BCL-2), and the tumor immune suppressive environment represent the most extensively validated drivers of PTX resistance. Correspondingly, the most promising predictive biomarkers for clinical evaluation include TUBB3 overexpression; P-gp/ABCB1 upregulation; activation of the PI3K/Akt, NF-кB, and MAPK signaling pathways; increases in key factors, e.g., BCL-2, FOXM1, and AURKA, and adaptive changed expression of epigenetic modified factors (ncRNAs, DNA methylation, and histone modifications); changed key factors or signaling pathways involved in cell plasticity, e.g., cell metabolism, protective autophagy, and immune response (PD-1/PD-L1); increased biomarkers for EMT and stemness in cancer cells; and dysregulated cell–cell communication factors.

However, numerous reversal strategies are conducted in the stage of preclinical research, although they show clinical promise. Actually, clinically actionable approaches remain limited to a few agents, such as epothilones for TUBB3-overexpressing tumors, venetoclax for BCL-2-dependent resistance, and decitabine for reversing hypermethylation-mediated gene silencing, as well as relacorilant combined with nab-paclitaxel for enhancing taxane efficacy in platinum-resistant ovarian cancer, although its role in directly reversing PTX resistance requires further validation.

A major translational barrier is the spatiotemporal heterogeneity of tumors, whereby multiple resistant subclones coexist and evolve under therapeutic pressure. This complexity is not recapitulated by conventional preclinical models. Furthermore, the extensive crosstalk among resistance pathways means that targeting a single node often leads to rapid compensatory adaptation. Addressing these challenges requires future research focused on dynamic, multi-omics-guided monitoring of resistance evolution and the rational design of multi-target combination regimens that can simultaneously disrupt multiple resistance mechanisms while leveraging advanced models like organoid-based platforms for personalized drug screening. Certainly, interdisciplinary integration with AI technologies could evaluate our research efficiency on identifying effective biomarkers and sifting appropriate drugs, providing clear research directions. New life or engineering technologies, e.g., organoids, could fill the translational gap from preclinical to clinical research. However, these new technologies themselves have many unsolved technological problems because they are not yet well established. In addition, the issue of PTX resistance involves more than just pharmacology, medicine, or biology. In fact, pharmaceutical formulation, administration methods, drug metabolism and interactions, and gut microbes and nutrition are also very important in PTX resistance. We still have a long way to go to solve the complicated problems of PTX resistance.

Nevertheless, this review systematically examines the core molecular mechanisms of PTX resistance, summarizes cutting-edge strategies to reverse chemoresistance and clinical translation, and discusses current challenges and future directions, thereby providing a theoretical framework for optimizing PTX-based regimens and overcoming clinical resistance. Ultimately, with precise molecular diagnosis, these dysfunctions could be targeted by synthetic small molecules or natural products or macromolecules to restore chemosensitivity.

## Figures and Tables

**Table 1:** Personalized molecular mechanisms of PTX resistance in different cancer types.

Resistance Drivers	Proposed Mechanisms	Types of PTX Resistance	Cross-Resistance to Other Compounds	Cancer Types	Experimental Model(s) and Evidence Level	References
Altered microtubule protein system	Mutations in beta-tubulin and reduced PTX binding to the site	Intrinsic resistance	Taxanes	NSCLC	Human biopsies from 49 stage IIIB and IV NSCLC patients	[189]
	Altered expression of beta tubulin isotypes or high expression of TUB33 increases microtubule dynamics and lowers the effect of PTX on microtublin	Intrinsic or acquired resistance	Taxanes	Ovarian cancer	Primary human tumor cell lines in vitro	[60]
	Changes in the expression of MAPs such as stathmin, tau, and MAP4 counteract the microtubule-stabilizing effect of PTX	Intrinsic or acquired resistance	Taxanes	Breast cancer	82 clinical cases	[190]
ABC transporter upregulation	Upregulation of ABCB1 level can promote drug efflux	Acquired resistance	Taxanes and other compounds	NSCLC	A549 cell line	[191]
	Increased ABCG2 can promote drug efflux	Acquired resistance	Taxanes and other compounds	Breast cancer	Taxol-resistant MCF-7 cell line	[192]
	Increased ABCC can promote drug efflux	Acquired resistance	Taxanes and other compounds	NSCLC and breast cancer	PTX-resistant breast and lung cancer cells	[71]
PI3K/Akt pathway activation	Abnormal activation of the PI3K/Akt pathway may promote cancer cell growth and promote PTX resistance	Acquired resistance	N/A	Ovarian cancer	Resistant cell lines and patient-derived cells	[78]
MAPK/ERK pathway activation	Activation of MAPK/EKR pathway attenuates apoptosis and promotes cancer cell growth and causes PTX resistance	Acquired resistance	N/A	NSCLC	Taxol-resistant NCI-H1299 cells	[193]
NF-κB pathway activation	Activation in the NF-κB pathway may promote cancer cell proliferation and migration and then induce PTX resistance	Acquired resistance	N/A	Breast cancer	Primary breast cancer cells	[95]
FOXM1 upregulation	Upregulation of FOXM1 can directly regulate MAPs and the cell cycle and promotes PTX resistance	Acquired resistance	Taxanes	Cervical cancer	Human cervical cancer tissues, cancer cell line, and CDX animal model	[102]
Aurora kinase activation	Aurora kinase overexpression can bypass the mitotic spindle assembly checkpoint and induce resistance to PTX	Acquired resistance	Taxanes	NSCLC	NSCLC and drug-resistant NSCLC xenograft tumor mice	[194]
BCL-2 upregulation	Increased BCL-2 attenuates PTX-induced apoptosis and mediates PTX resistance	Acquired resistance	N/A	Breast cancer	MDA-MB-231 TNBC cells	[195]
GR activation	GR activation can modulate apoptotic pathway and promote PTX resistance	Acquired resistance	N/A	Ovarian cancer	Phase 3 clinical trial	[119]
Autophagy induction	Increased protective autophagy promotes cancer cell survival and PTX resistance	Acquired resistance	N/A	Breast cancer	Cancer cell lines and xenograft model	[188]
Cell metabolism adaption	Elevated aerobic glycolysis contributes to cancer proliferation and causes PTX resistance	Acquired resistance	N/A	Ovarian cancer	Ovarian tissues and cells and xenograft model	[196]
Epigenetic modifications	Abnormal ncRNA expression can regulate cancer-associated genes and related targets and then promote PTX resistance through various mechanisms	Acquired resistance	N/A	Breast cancer; Ovarian cancer; Gastric cancer	Breast cancer tissues and cell lines; ovarian cancer cell lines; gastric cancer cell lines and tissues	[145,156,162,163]
	DNA methylation regulates gene expression and promotes PTX resistance	Acquired resistance	N/A	Breast cancer	MCF-7/Taxol cells; breast cancer cells	[45,168,169]
	Histone modifications and chromatin remodeling regulate oncogene expression and promote PTX resistance	Acquired resistance	N/A	Ovarian cancer Breast cancer	PTX-resistant ovarian, breast cancer cell lines	[69,170,171,197]
Chromosomal instability	CIN can induce G1 phase arrest and structural alterations in chromosome and cause resistance to anticancer therapies	Acquired resistance	N/A	Breast cancer	Cancer cell lines	[173,176]
Cell plasticity	Cells with mesenchymal features are more prone to entering a quiescent or slow-cycling state and become DTPs	Acquired resistance	N/A	Ovarian cancer	Cancer cell lines	[186]
	Cells with stemness properties are also more prone to becoming DTPs	Acquired resistance	N/A	Breast cancer	PTX-resistant cell lines	[187]
TME remodeling	Hypoxia-induced gp96 degrades p53, reducing PTX sensitivity, and activates cell pyroptosis in CD8^+^ T cells	Acquired resistance	N/A	Breast cancer	Cancer cell lines	[182]
	Immunosuppressive remodeling (e.g., increased Tregs and M2 macrophage polarization) dampens antitumor immunity	Acquired resistance	N/A	Melanoma	Cell lines in vitro and a metastatic melanoma mouse model	[55,58,198]
	Functional shifts in CAF subtype mediated by JAK2 activation	Acquired resistance	N/A	Breast cancer	Patient-derived xenograft (PDX) models and TNBC cell lines	[183]
	Inflammation-stimulated CCL2 overexpression activates PI3K/Akt in cancer cells and promotes M2 phenotype	Acquired resistance	N/A	Ovarian cancer	Human ovarian cancer cells	[184]
	PTX activates TLR4, promoting angiogenesis and lymphangiogenesis	Acquired resistance	N/A	N/A	Review of mechanistic concepts	[57]
	Drug-resistant cells deliver miR-155-5p to sensitive cells via sEVs	Acquired resistance	N/A	Gastric cancer	PTX-resistant and -sensitive gastric cancer cells	[155]

**Table 2:** Summary of strategies for reversing PTX resistance.

Strategy Category	Specific Targets/Pathways	Reversal Strategies/Medications	Drug Properties	Clinical or Preclinical Stage	Joint Program	References
Microtubule-associated proteins	Targets microtube proteins but not affected by increased TUBB3	Epothilones (e.g., Ixabepilone and Patupilone)	Natural products	FDA-approved drug (Ixabepilone) in taxane-resistant setting (TNBC) Phase III ovarian cancer (Patupilone) Negative	Not combined with PTX N/A	[200,219,220]
External pump suppression	P-gp/ABCB1	Valspodar (PSC-833)	Small-molecule inhibitors	Clinical pharmacokinetic trial; phase III clinical study (failure due to efficacy and toxicity)	Combined with PTX	[221,222]
	P-gp/ABCB1	Polyphyllin H	Natural products	Preclinical	Combined with PTX	[68]
	P-gp/ABCB1	Apigenin	Natural flavonoids	Preclinical	Combined with PTX	[66]
	P-gp/ABCB1 + PI3K/mTOR	DHW-221 (dual-target PI3K/mTOR inhibitor)	Small-molecule inhibitors	Preclinical	Combined with PTX	[73]
	P-gp + CD47	non-humanized chimeric bispecific antibody	Antibody-based therapeutics	Preclinical	Combined with PTX	[67]
PI3K/Akt pathway	PI3K/Akt/NF-κB	Cajanol	Natural products	Preclinical	Combined with PTX	[77]
	PI3K/Akt/mTOR	RTEs	Natural products	Preclinical	Combined with PTX (TNBC)	[201]
MAPK/ERK pathway	MEK/ERK/Mnk1/eIF4E	U0126 (MEK inhibitor)	Small-molecule inhibitors	Preclinical	Combined with PTX	[82]
	MAPK→P-gp	Ganoderma lucidum extract	Natural products	Preclinical	Combined with PTX	[86]
	MEK	Cobimetinib	Small-molecule inhibitors	Phase II clinical (negative results)	Combined with PTX (phase II; negative)	[88,223]
NF-κB pathway	NF-κB	ALLN (proteasome inhibitor)	Small-molecule inhibitors	Preclinical	Combined with PTX	[95]
	NF-κB	VOA	Natural products	Preclinical	Combined with PTX (ovarian cancer)	[202]
AURKA	Aurora kinase A	PEITC	Natural products	Preclinical	Combined with PTX (breast cancer)	[203]
	Aurora kinase A	CYC3 (new AK-A inhibitor)	Small-molecule inhibitors	Preclinical	Combined with PTX (pancreatic Cancer)	[106]
FOXM1	FOXM1/Survivin (not direct action)	Domatinostat	Class I HDAC inhibitor	Preclinical	Combination of PTX and Domatinostat (pancreatic cancer)	[204,205]
BCL-2 family	Bcl-2	Venetoclax	Small-molecule inhibitors	Preclinical	Combined with nab-PTX (Ewing sarcoma, PDX model)	[206]
GR signaling	Glucocorticoid receptor (GR)	Relacorilant	Selective GR antagonist	Phase III completed (ROSELLA trial)	Combined with nab-PTX (PROC)	[209,210,224]
Autophagy regulation	PI3K/AKT inhibitor-induced autophagy	Ipatasertib and Taselisib	Small-molecule inhibitors	Preclinical	Combined with taxanes (TNBC)	[136]
	Autophagy	Chloroquine	Small-molecule inhibitors	Preclinical	Combined with PTX (NSCLC)	[211]
Epigenetics	DNMT1/KLF4	DIM	Natural products	Preclinical	Combined with PTX (breast cancer)	[168]
	DNMT	Decitabine	Small-molecule nucleoside analog	Preclinical	Combined with PTX (breast cancer)	[45]
Chromosomal instability	STING	S-72 (tubulin inhibitor)	Small-molecule inhibitors	Preclinical	Combined with PTX (breast cancer)	[212]
	SIK2	MRIA9 (SIK2 inhibitor)	Small-molecule inhibitors	Preclinical	Combined with PTX (ovarian cancer)	[213]
Metabolic reprogramming	Fatty acid metabolism	FV-429	Natural product derivatives	Preclinical	Combined with PTX	[215]
	HK2	HK2 inhibitor	Small-molecule inhibitors	Preclinical	Combined with PTX (ovarian cancer)	[214]
TME	PD-L1	Anti-PD-L1 blocking antibody	Antibodies	In patients with TNBC, atezolizumab in combination with nab-paclitaxel-based chemotherapy significantly improved pathological complete response rates with an acceptable safety profile (phase 3 trial)	Combined with PTX (TNBC)	[216]
	TGF-β/SMAD2	Alpha-hederin (sEV-mediated miRNAs)	Natural products	Preclinical	Combined with PTX (NSCLC)	[217]
	Multi-target (TME + tumor cells)	Rg3-PTX-LPs	Nano-delivery system	Preclinical	PTX-loaded liposomes (breast cancer)	[218]

## Data Availability

No new data were created or analyzed in this study. Data sharing is not applicable to this article.

## References

[1] Zafar A., Khatoon S., Khan M.J., Abu J., Naeem A. (2025). Advancements and Limitations in Traditional Anti-Cancer Therapies: A Comprehensive Review of Surgery, Chemotherapy, Radiation Therapy, and Hormonal Therapy. Discov. Oncol..

[2] Lahiri A., Maji A., Potdar P.D., Singh N., Parikh P., Bisht B., Mukherjee A., Paul M.K. (2023). Lung Cancer Immunotherapy: Progress, Pitfalls, and Promises. Mol. Cancer.

[3] Biller L.H., Schrag D. (2021). Diagnosis and Treatment of Metastatic Colorectal Cancer: A Review. JAMA J. Am. Med. Assoc..

[4] Harbeck N., Gnant M. (2017). Breast Cancer. Lancet.

[5] Kaur R., Bhardwaj A., Gupta S. (2023). Cancer Treatment Therapies: Traditional to Modern Approaches to Combat Cancers. Mol. Biol. Rep..

[6] Dumontet C., Reichert J.M.M., Senter P.D.D., Lambert J.M.M., Beck A. (2023). Antibody-Drug Conjugates Come of Age in Oncology. Nat. Rev. Drug Discov..

[7] Chirnomas D., Hornberger K.R., Crews C.M. (2023). Protein Degraders Enter the Clinic—A New Approach to Cancer Therapy. Nat. Rev. Clin. Oncol..

[8] Mottamal M., Zheng S., Huang T.L., Wang G. (2015). Histone Deacetylase Inhibitors in Clinical Studies as Templates for New Anticancer Agents. Molecules.

[9] June C.H., O’Connor R.S., Kawalekar O.U., Ghassemi S., Milone M.C. (2018). CAR T Cell Immunotherapy for Human Cancer. Science.

[10] Johnson D.E., O’Keefe R.A., Grandis J.R. (2018). Targeting the IL-6/JAK/STAT3 Signalling Axis in Cancer. Nat. Rev. Clin. Oncol..

[11] Perez-Herrero E., Fernandez-Medarde A. (2015). Advanced Targeted Therapies in Cancer: Drug Nanocarriers, the Future of Chemotherapy. Eur. J. Pharm. Biopharm..

[12] Lim P.T., Goh B.H., Lee W.L. (2022). Taxol: Mechanisms of Action against Cancer, an Update with Current Research. Paclitaxel.

[13] Alalawy A.I. (2024). Key Genes and Molecular Mechanisms Related to Paclitaxel Resistance. Cancer Cell Int..

[14] Castaneda M., den Hollander P., Kuburich N.A., Rosen J.M., Mani S.A. (2022). Mechanisms of Cancer Metastasis. Semin. Cancer Biol..

[15] Al-Mahayri Z.N., AlAhmad M.M., Ali B.R. (2021). Current Opinion on the Pharmacogenomics of Paclitaxel-Induced Toxicity. Expert Opin. Drug Metab. Toxicol..

[16] Gallego-Jara J., Lozano-Terol G., Sola-Martínez R.A., Cánovas-Díaz M., Puente T.d.D. (2020). A Compressive Review about Taxol^®^: History and Future Challenges. Molecules.

[17] Zhao S., Tang Y., Wang R., Najafi M. (2022). Mechanisms of Cancer Cell Death Induction by Paclitaxel: An Updated Review. Apoptosis.

[18] Abouzeid H.A., Kassem L., Liu X., Abuelhana A. (2025). Paclitaxel Resistance in Breast Cancer: Current Challenges and Recent Advanced Therapeutic Strategies. Cancer Treat. Res. Commun..

[19] Rivera E., Gomez H. (2010). Chemotherapy Resistance in Metastatic Breast Cancer: The Evolving Role of Ixabepilone. Breast Cancer Res. BCR.

[20] Kavallaris M. (2010). Microtubules and Resistance to Tubulin-Binding Agents. Nat. Rev. Cancer.

[21] Zhou J., Giannakakou P. (2005). Targeting Microtubules for Cancer Chemotherapy. Curr. Med. Chem.-Anti-Cancer Agents.

[22] Sati P., Sharma E., Dhyani P., Attri D.C., Rana R., Kiyekbayeva L., Büsselberg D., Samuel S.M., Sharifi-Rad J. (2024). Paclitaxel and Its Semi-Synthetic Derivatives: Comprehensive Insights into Chemical Structure, Mechanisms of Action, and Anticancer Properties. Eur. J. Med. Res..

[23] Zhu L., Chen L. (2019). Progress in Research on Paclitaxel and Tumor Immunotherapy. Cell. Mol. Biol. Lett..

[24] Foley E.A., Kapoor T.M. (2013). Microtubule Attachment and Spindle Assembly Checkpoint Signalling at the Kinetochore. Nat. Rev. Mol. Cell Biol..

[25] Ojeda-Lopez M.A., Needleman D.J., Song C., Ginsburg A., Kohl P.A., Li Y., Miller H.P., Wilson L., Raviv U., Choi M.C. (2014). Transformation of Taxol-Stabilized Microtubules into Inverted Tubulin Tubules Triggered by a Tubulin Conformation Switch. Nat. Mater..

[26] Nawara H.M., Afify S.M., Hassan G., Zahra M.H., Seno A., Seno M. (2021). Paclitaxel-Based Chemotherapy Targeting Cancer Stem Cells from Mono- to Combination Therapy. Biomedicines.

[27] Ferlini C., Raspaglio G., Mozzetti S., Distefano M., Filippetti F., Martinelli E., Ferrandina G., Gallo D., Ranelletti F.O., Scambia G. (2003). Bcl-2 down-Regulation Is a Novel Mechanism of Paclitaxel Resistance. Mol. Pharmacol..

[28] André N., Braguer D., Brasseur G., Gonçalves A., Lemesle-Meunier D., Guise S., Jordan M.A., Briand C. (2000). Paclitaxel Induces Release of Cytochrome c from Mitochondria Isolated from Human Neuroblastoma Cells’. Cancer Res..

[29] von Haefen C., Wieder T., Essmann F., Schulze-Osthoff K., Dörken B., Daniel P.T. (2003). Paclitaxel-Induced Apoptosis in BJAB Cells Proceeds via a Death Receptor-Independent, Caspases-3/-8-Driven Mitochondrial Amplification Loop. Oncogene.

[30] Yu Y., Zhu J., Li J., Tang J. (2024). The Mechanism of Paclitaxel Induced Damage on Placental Trophoblast Cells. BMC Pregnancy Childbirth.

[31] Li M., Yin L., Wu L., Zhu Y., Wang X. (2020). Paclitaxel Inhibits Proliferation and Promotes Apoptosis through Regulation ROS and Endoplasmic Reticulum Stress in Osteosarcoma Cell. Mol. Cell. Toxicol..

[32] Janczar S., Nautiyal J., Xiao Y., Curry E., Sun M., Zanini E., Paige A.J., Gabra H. (2017). WWOX Sensitises Ovarian Cancer Cells to Paclitaxel via Modulation of the ER Stress Response. Cell Death Dis..

[33] Mohiuddin M., Kasahara K. (2021). The Mechanisms of the Growth Inhibitory Effects of Paclitaxel on Gefitinib-Resistant Non-Small Cell Lung Cancer Cells. Cancer Genom. Proteom..

[34] Liebl M.C., Hofmann T.G. (2021). The Role of P53 Signaling in Colorectal Cancer. Cancers.

[35] Wu W., Wei T., Li Z., Zhu J. (2021). P53-Dependent Apoptosis Is Essential for the Antitumor Effect of Paclitaxel Response to DNA Damage in Papillary Thyroid Carcinoma. Int. J. Med. Sci..

[36] Noh H.-J., Kim K.-A., Kim K.-C. (2014). P53 Down-Regulates SETDB1 Gene Expression during Paclitaxel Induced-Cell Death. Biochem. Biophys. Res. Commun..

[37] Xu J., Su C., Zhao F., Tao J., Hu D., Shi A., Pan J., Zhang Y. (2018). Paclitaxel Promotes Lung Cancer Cell Apoptosis via MEG3-P53 Pathway Activation. Biochem. Biophys. Res. Commun..

[38] Park J.-H., Park S.-A., Lee Y.-J., Park H.-W., Oh S.-M. (2020). PBK Attenuates Paclitaxel-Induced Autophagic Cell Death by Suppressing P53 in H460 Non-Small-Cell Lung Cancer Cells. FEBS Open Bio.

[39] Rishi G., Huang G., Subramaniam V.N. (2021). Cancer: The Role of Iron and Ferroptosis. Int. J. Biochem. Cell Biol..

[40] Dixon S.J., Lemberg K.M., Lamprecht M.R., Skouta R., Zaitsev E.M., Gleason C.E., Patel D.N., Bauer A.J., Cantley A.M., Yang W.S. (2012). Ferroptosis: An Iron-Dependent Form of Non-Apoptotic Cell Death. Cell.

[41] Li X., Gui B., Yu Y., Liu F. (2024). Paclitaxel Inhibits Thyroid Cancer by Regulating AMPK/mTOR and Promoting Ferroptosis. J. Biomed. Nanotechnol..

[42] Qiu Y., Yu Q., Ji M., Zhang Z., Kang L., Fu Y., Feng M. (2021). Activation Ferroptosis Enhanced the Therapy Sensitivity of TNBC to Paclitaxel via NCOA4 Mediated Ferritinophagy. https://assets-eu.researchsquare.com/files/rs-360631/v1/fea159f9-028a-4b34-830a-c969e70e7f00.pdf?c=1631880687.

[43] Zhang C.-C., Li C.-G., Wang Y.-F., Xu L.-H., He X.-H., Zeng Q.-Z., Zeng C.-Y., Mai F.-Y., Hu B., Ouyang D.-Y. (2019). Chemotherapeutic Paclitaxel and Cisplatin Differentially Induce Pyroptosis in A549 Lung Cancer Cells via Caspase-3/GSDME Activation. Apoptosis Int. J. Program. Cell Death.

[44] Wang X., Li H., Li W., Xie J., Wang F., Peng X., Song Y., Tan G. (2020). The Role of Caspase-1/GSDMD-Mediated Pyroptosis in Taxol-Induced Cell Death and a Taxol-Resistant Phenotype in Nasopharyngeal Carcinoma Regulated by Autophagy. Cell Biol. Toxicol..

[45] Gong W., Fang P., Leng M., Shi Y. (2023). Promoting GSDME Expression through DNA Demethylation to Increase Chemosensitivity of Breast Cancer MCF-7/Taxol Cells. PLoS ONE.

[46] Usman R.M., Razzaq F., Akbar A., Farooqui A.A., Iftikhar A., Latif A., Hassan H., Zhao J., Carew J.S., Nawrocki S.T. (2021). Role and Mechanism of Autophagy-Regulating Factors in Tumorigenesis and Drug Resistance. Asia-Pac. J. Clin. Oncol..

[47] Eum K.-H., Lee M. (2011). Crosstalk between Autophagy and Apoptosis in the Regulation of Paclitaxel-Induced Cell Death in v-Ha-Ras-Transformed Fibroblasts. Mol. Cell. Biochem..

[48] Lee Y., Na J., Lee M.S., Cha E.Y., Sul J.Y., Park J.B., Lee J.S. (2018). Combination of Pristimerin and Paclitaxel Additively Induces Autophagy in Human Breast Cancer Cells via ERK1/2 Regulation. Mol. Med. Rep..

[49] Yu Y.-F., Hu P.-C., Wang Y., Xu X.-L., Rushworth G.M., Zhang Z., Wei L., Zhang J.-W. (2017). Paclitaxel Induces Autophagy in Gastric Cancer BGC823 Cells. Ultrastruct. Pathol..

[50] Xie Q., Chen Y., Tan H., Liu B., Zheng L.-L., Mu Y. (2021). Targeting Autophagy with Natural Compounds in Cancer: A Renewed Perspective from Molecular Mechanisms to Targeted Therapy. Front. Pharmacol..

[51] Xi G., Hu X., Wu B., Jiang H., Young C.Y.F., Pang Y., Yuan H. (2011). Autophagy Inhibition Promotes Paclitaxel-Induced Apoptosis in Cancer Cells. Cancer Lett..

[52] Khing T.M., Choi W.S., Kim D.M., Po W.W., Thein W., Shin C.Y., Sohn U.D. (2021). The Effect of Paclitaxel on Apoptosis, Autophagy and Mitotic Catastrophe in AGS Cells. Sci. Rep..

[53] Javeed A., Ashraf M., Riaz A., Ghafoor A., Afzal S., Mukhtar M.M. (2009). Paclitaxel and Immune System. Eur. J. Pharm. Sci. Off. J. Eur. Fed. Pharm. Sci..

[54] Vicari A.P., Luu R., Zhang N., Patel S., Makinen S.R., Hanson D.C., Weeratna R.D., Krieg A.M. (2009). Paclitaxel Reduces Regulatory T Cell Numbers and Inhibitory Function and Enhances the Anti-Tumor Effects of the TLR9 Agonist PF-3512676 in the Mouse. Cancer Immunol. Immunother. CII.

[55] Wanderley C.W., Colón D.F., Luiz J.P.M., Oliveira F.F., Viacava P.R., Leite C.A., Pereira J.A., Silva C.M., Silva C.R., Silva R.L. (2018). Paclitaxel Reduces Tumor Growth by Reprogramming Tumor-Associated Macrophages to an M1 Profile in a TLR4-Dependent Manner. Cancer Res..

[56] Muraro E., Comaro E., Talamini R., Turchet E., Miolo G., Scalone S., Militello L., Lombardi D., Spazzapan S., Perin T. (2015). Improved Natural Killer Cell Activity and Retained Anti-Tumor CD8+ T Cell Responses Contribute to the Induction of a Pathological Complete Response in HER2-Positive Breast Cancer Patients Undergoing Neoadjuvant Chemotherapy. J. Transl. Med..

[57] Volk-Draper L., Hall K., Griggs C., Rajput S., Kohio P., DeNardo D., Ran S. (2014). Paclitaxel Therapy Promotes Breast Cancer Metastasis in a TLR4-Dependent Manner. Cancer Res..

[58] Oweida A., Hararah M.K., Phan A., Binder D., Bhatia S., Lennon S., Bukkapatnam S., Van Court B., Uyanga N., Darragh L. (2018). Resistance to Radiotherapy and PD-L1 Blockade Is Mediated by TIM-3 Upregulation and Regulatory T-Cell Infiltration. Clin. Cancer Res. Off. J. Am. Assoc. Cancer Res..

[59] Orr G.A., Verdier-Pinard P., McDaid H., Horwitz S.B. (2003). Mechanisms of Taxol Resistance Related to Microtubules. Oncogene.

[60] Kavallaris M., Kuo D.Y., Burkhart C.A., Regl D.L., Norris M.D., Haber M., Horwitz S.B. (1997). Taxol-Resistant Epithelial Ovarian Tumors Are Associated with Altered Expression of Specific Beta-Tubulin Isotypes. J. Clin. Investig..

[61] McGrail D.J., Khambhati N.N., Qi M.X., Patel K.S., Ravikumar N., Brandenburg C.P., Dawson M.R. (2015). Alterations in Ovarian Cancer Cell Adhesion Drive Taxol Resistance by Increasing Microtubule Dynamics in a FAK-Dependent Manner. Sci. Rep..

[62] Liu J., Li J., Wang K., Liu H., Sun J., Zhao X., Yu Y., Qiao Y., Wu Y., Zhang X. (2021). Aberrantly High Activation of a FoxM1–STMN1 Axis Contributes to Progression and Tumorigenesis in FoxM1-Driven Cancers. Signal Transduct. Target. Ther..

[63] Gillet J.-P., Gottesman M.M. (2011). Advances in the Molecular Detection of ABC Transporters Involved in Multidrug Resistance in Cancer. Curr. Pharm. Biotechnol..

[64] Gillet J.-P., Gottesman M.M. (2010). Mechanisms of Multidrug Resistance in Cancer. Methods Mol. Biol..

[65] Stasiak P., Sopel J., Lipowicz J.M., Rawłuszko-Wieczorek A.A., Sterzyńska K., Korbecki J., Januchowski R. (2025). Elacridar Reverses P-Gp-Mediated Drug Resistance in Ovarian Cancer Cells in 2D and 3D Culture Models. Int. J. Mol. Sci..

[66] Padder K.A. (2025). Apigenin Modulates Expression Pattern of Cancer Multidrug Resistance Proteins in Non-Small Lung Cancer Cell Line. J. Biochem. Mol. Toxicol..

[67] Briante R., Zhai Q., Mohanty S., Zhang P., O’Connor A., Misker H., Wang W., Tan C., Abuhay M., Morgan J. (2025). Successful Targeting of Multidrug-Resistant Tumors with Bispecific Antibodies. mAbs.

[68] Ye Z., Hong C., Jiang M., Zou W., Ren Y., Li M., Xue X., Xie X., Zhang T., Ding Y. (2025). Polyphyllin H Reverses Paclitaxel Resistance in Breast Cancer by Binding Membrane Cholesterol to Inhibit Both ABCB1 and ABCC3. Pharmaceuticals.

[69] Kohli A., Huang S.-L., Chang T.-C., Chao C.C.-K., Sun N.-K. (2022). H1.0 Induces Paclitaxel-Resistance Genes Expression in Ovarian Cancer Cells by Recruiting GCN5 and Androgen Receptor. Cancer Sci..

[70] Gronkowska K., Michlewska S., Robaszkiewicz A. (2023). Activity of Lysosomal ABCC3, ABCC5 and ABCC10 Is Responsible for Lysosomal Sequestration of Doxorubicin and Paclitaxel-Oregongreen488 in Paclitaxel-Resistant Cancer Cell Lines. Cell Physiol. Biochem. Int. J. Exp. Cell. Physiol. Biochem. Pharmacol..

[71] Gronkowska K., Kołacz-Milewska K., Michlewska S., Płoszaj T., Borowiec M., Robaszkiewicz A. (2025). HIF1A, BRG1, and P300 Interaction Confers Paclitaxel-Induced Drug Resistance by Enabling the Overexpression of ABCC Genes. Mol. Ther. Oncol..

[72] Shuai Y., Ma Z., Yue J., Li C., Ju J., Wang X., Qian H., Yuan P. (2025). MNX1-AS1 Suppresses Chemosensitivity by Activating the PI3K/AKT Pathway in Breast Cancer. Int. J. Biol. Sci..

[73] Liu M., Xu C., Qin X., Liu W., Li D., Jia H., Gao X., Wu Y., Wu Q., Xu X. (2022). DHW-221, a Dual PI3K/mTOR Inhibitor, Overcomes Multidrug Resistance by Targeting P-Glycoprotein (P-Gp/ABCB1) and Akt-Mediated FOXO3a Nuclear Translocation in Non-Small Cell Lung Cancer. Front. Oncol..

[74] Yang J., Nie J., Ma X., Wei Y., Peng Y., Wei X. (2019). Targeting PI3K in Cancer: Mechanisms and Advances in Clinical Trials. Mol. Cancer.

[75] Zi D., Li Q., Xu C., Zhou Z.-W., Song G.-B., Hu C.-B., Wen F., Yang H.-L., Nie L., Zhao X. (2022). CXCR4 Knockdown Enhances Sensitivity of Paclitaxel via the PI3K/Akt/mTOR Pathway in Ovarian Carcinoma. Aging.

[76] Chen M., Lu J., Wei W., Lv Y., Zhang X., Yao Y., Wang L., Ling T., Zou X. (2018). Effects of Proton Pump Inhibitors on Reversing Multidrug Resistance via Downregulating V-ATPases/PI3K/Akt/mTOR/HIF-1α Signaling Pathway through TSC1/2 Complex and Rheb in Human Gastric Adenocarcinoma Cells in Vitro and in Vivo. OncoTargets Ther..

[77] Sui M., Yang H., Guo M., Li W., Gong Z., Jiang J., Li P. (2021). Cajanol Sensitizes A2780/Taxol Cells to Paclitaxel by Inhibiting the PI3K/Akt/NF-κB Signaling Pathway. Front. Pharmacol..

[78] Raivola J., Rantanen F., Dini A., Piki E., Barker H., Multamäki E., Bentz F., Sunkel P., Kiiski A.-M., Paavolainen L. (2026). ROR1-PI3K/AKT Signaling Drives Adaptive Resistance to Cell Cycle Blockade in TP53 Mutated Ovarian Cancer. Cell Death Dis..

[79] Lahtinen A., Lavikka K., Virtanen A., Li Y., Jamalzadeh S., Skorda A., Lauridsen A.R., Zhang K., Marchi G., Isoviita V.-M. (2023). Evolutionary States and Trajectories Characterized by Distinct Pathways Stratify Patients with Ovarian High Grade Serous Carcinoma. Cancer Cell.

[80] Dini A., Barker H., Piki E., Sharma S., Raivola J., Murumägi A., Ungureanu D. (2025). A Multiplex Single-Cell RNA-Seq Pharmacotranscriptomics Pipeline for Drug Discovery. Nat. Chem. Biol..

[81] Zhang Q., Xu B., Hu F., Chen X., Liu X., Zhang Q., Zuo Y. (2021). Tenascin C Promotes Glioma Cell Malignant Behavior and Inhibits Chemosensitivity to Paclitaxel via Activation of the PI3K/AKT Signaling Pathway. J. Mol. Neurosci..

[82] Liu S., Zha J., Lei M. (2018). Inhibiting ERK/Mnk/eIF4E Broadly Sensitizes Ovarian Cancer Response to Chemotherapy. Clin. Transl. Oncol..

[83] Kudaravalli S., Hollander P.d., Mani S.A. (2022). Role of P38 MAP Kinase in Cancer Stem Cells and Metastasis. Oncogene.

[84] Martínez-Limón A., Joaquin M., Caballero M., Posas F., de Nadal E. (2020). The P38 Pathway: From Biology to Cancer Therapy. Int. J. Mol. Sci..

[85] Salaroglio I.C., Mungo E., Gazzano E., Kopecka J., Riganti C. (2019). ERK Is a Pivotal Player of Chemo-Immune-Resistance in Cancer. Int. J. Mol. Sci..

[86] Jiao C., Qiu J., Gong C., Li X., Liang H., He C., Cen S., Xie Y. (2025). Ganoderma Lucidum Extract Reverses Multidrug Resistance in Breast Cancer Cells through Inhibiting ATPase Activity of the P-Glycoprotein via MAPK/ERK Signaling Pathway. Exp. Cell Res..

[87] Yan F., Wang M., Li J., Cheng H., Su J., Wang X., Wu H., Xia L., Li X., Chang H.C. (2012). Gambogenic Acid Induced Mitochondrial-Dependent Apoptosis and Referred to Phospho-Erk1/2 and Phospho-P38 MAPK in Human Hepatoma HepG2 Cells. Environ. Toxicol. Pharmacol..

[88] Liu Y., Zhao R., Qin X., Mao X., Li Q., Fang S. (2022). Cobimetinib Sensitizes Cervical Cancer to Paclitaxel via Suppressing Paclitaxel-Induced ERK Activation. Pharmacology.

[89] Lin H., Hu B., He X., Mao J., Wang Y., Wang J., Zhang T., Zheng J., Peng Y., Zhang F. (2020). Overcoming Taxol-Resistance in A549 Cells: A Comprehensive Strategy of Targeting P-Gp Transporter, AKT/ERK Pathways, and Cytochrome P450 Enzyme CYP1B1 by 4-Hydroxyemodin. Biochem. Pharmacol..

[90] Guo Q., Qiu Y., Liu Y., He Y., Zhang G., Du Y., Yang C., Gao F. (2022). Cell Adhesion Molecule CD44v10 Promotes Stem-like Properties in Triple-Negative Breast Cancer Cells via Glucose Transporter GLUT1-Mediated Glycolysis. J. Biol. Chem..

[91] Yu H., Lin L., Zhang Z., Zhang H., Hu H. (2020). Targeting NF-κB Pathway for the Therapy of Diseases: Mechanism and Clinical Study. Signal Transduct. Target. Ther..

[92] Karin M., Cao Y., Greten F.R., Li Z.-W. (2002). NF-κB in Cancer: From Innocent Bystander to Major Culprit. Nat. Rev. Cancer.

[93] Aggarwal B.B., Sung B. (2011). NF-κB in Cancer: A Matter of Life and Death. Cancer Discov..

[94] Xu H., Du Z., Li Z., Liu X., Li X., Zhang X., Ma J. (2024). MUC1-EGFR Crosstalk with IL-6 by Activating NF-κB and MAPK Pathways to Regulate the Stemness and Paclitaxel-Resistance of Lung Adenocarcinoma. Ann. Med..

[95] Esparza-López J., Longoria O., De La Cruz-Escobar E.N., Garibay-Díaz J.C., León-Rodríguez E., De Jesús Ibarra-Sánchez M. (2022). Paclitaxel Resistance Is Mediated by NF-κB on Mesenchymal Primary Breast Cancer Cells. Oncol. Lett..

[96] Cong Y., Cui Y., Zhu S., Cao J., Zou H., Martin T.A., Qiao G., Jiang W., Yu Z. (2020). Tim-3 Promotes Cell Aggressiveness and Paclitaxel Resistance through NF-κB/STAT3 Signalling Pathway in Breast Cancer Cells. Chin. J. Cancer Res..

[97] Lai T.-C., Fang C.-Y., Jan Y.-H., Hsieh H.-L., Yang Y.-F., Liu C.-Y., Chang P.M.-H., Hsiao M. (2020). Kinase shRNA Screening Reveals That TAOK3 Enhances Microtubule-Targeted Drug Resistance of Breast Cancer Cells via the NF-κB Signaling Pathway. Cell Commun. Signal..

[98] Tan X.-Q., Guo A.-Y., Zheng L.-F., Xiong J. (2025). Research Progress on FOXM1 in Ovarian Cancer Diagnosis and Therapeutics. Front. Oncol..

[99] Wierstra I. (2013). FOXM1 (Forkhead Box M1) in Tumorigenesis: Overexpression in Human Cancer, Implication in Tumorigenesis, Oncogenic Functions, Tumor-Suppressive Properties, and Target of Anticancer Therapy. Advances in Cancer Research.

[100] Zhang Z., Li M., Sun T., Zhang Z., Liu C. (2023). FOXM1: Functional Roles of FOXM1 in Non-Malignant Diseases. Biomolecules.

[101] Huang X., Zhang X., Machireddy N., Evans C.E., Trewartha S.D., Hu G., Fang Y., Mutlu G.M., Wu D., Zhao Y.-Y. (2023). Endothelial FoxM1 Reactivates Aging-Impaired Endothelial Regeneration for Vascular Repair and Resolution of Inflammatory Lung Injury. Sci. Transl. Med..

[102] Tang Q., Xu A., Yang Y., Zhang Y., Sun J. (2023). FOXM1 Contributes to Chemotherapy Sensitivity in Cervical Cancer by Regulating TTK. Discov. Med..

[103] Zhang X., Huang C., Yuan Y., Jin S., Zhao J., Zhang W., Liang H., Chen X., Zhang B. (2022). FOXM1-Mediated Activation of Phospholipase D1 Promotes Lipid Droplet Accumulation and Reduces ROS to Support Paclitaxel Resistance in Metastatic Cancer Cells. Free Radic. Biol. Med..

[104] Zhao C., Chen H.-Y., Zhao F., Feng H.-J., Su J.-P. (2021). Acylglycerol Kinase Promotes Paclitaxel Resistance in Nasopharyngeal Carcinoma Cells by Regulating FOXM1 via the JAK2/STAT3 Pathway. Cytokine.

[105] Hou Y., Dong Z., Zhong W., Yin L., Li X., Kuerban G., Huang H. (2022). FOXM1 Promotes Drug Resistance in Cervical Cancer Cells by Regulating ABCC5 Gene Transcription. BioMed Res. Int..

[106] Lin Y., Richards F.M., Krippendorff B.-F., Bramhall J.L., Harrington J.A., Bapiro T.E., Robertson A., Zheleva D., Jodrell D.I. (2012). Paclitaxel and CYC3, an Aurora Kinase a Inhibitor, Synergise in Pancreatic Cancer Cells but Not Bone Marrow Precursor Cells. Br. J. Cancer.

[107] Gautschi O., Heighway J., Mack P.C., Purnell P.R., Lara P.N., Gandara D.R. (2008). Aurora Kinases as Anticancer Drug Targets. Clin. Cancer Res. Off. J. Am. Assoc. Cancer Res..

[108] Anand S., Penrhyn-Lowe S., Venkitaraman A.R. (2003). AURORA-a Amplification Overrides the Mitotic Spindle Assembly Checkpoint, Inducing Resistance to Taxol. Cancer Cell.

[109] Li Y., Tang K., Zhang H., Zhang Y., Zhou W., Chen X. (2011). Function of Aurora Kinase a in Taxol-Resistant Breast Cancer and Its Correlation with P-Gp. Mol. Med. Rep..

[110] Ning B., Liu C., Kucukdagli A.C., Zhang J., Jing H., Zhou Z., Zhang Y., Dong Y., Chen Y., Guo H. (2025). Proteomic Profiling Identifies Upregulation of Aurora Kinases Causing Resistance to Taxane-Type Chemotherapy in Triple Negative Breast Cancer. Sci. Rep..

[111] Youle R.J., Strasser A. (2008). The BCL-2 Protein Family: Opposing Activities That Mediate Cell Death. Nat. Rev. Mol. Cell Biol..

[112] Raab M., Kobayashi N.F., Becker S., Kurunci-Csacsko E., Krämer A., Strebhardt K., Sanhaji M. (2020). Boosting the Apoptotic Response of High-Grade Serous Ovarian Cancers with CCNE1 Amplification to Paclitaxel in Vitro by Targeting APC/C and the pro-Survival Protein MCL-1. Int. J. Cancer.

[113] Liu D., Qin X., Sun Z., Hou S., Lv Q. (2020). Low DEDD Expression in Breast Cancer Cells Indicates Higher Sensitivity to the Bcl-2-Specific Inhibitor ABT-199. Biochem. Biophys. Res. Commun..

[114] Yang J., Li N., Zhao X., Guo W., Wu Y., Nie C., Yuan Z. (2024). WP1066, a Small Molecule Inhibitor of STAT3, Chemosensitizes Paclitaxel-Resistant Ovarian Cancer Cells to Paclitaxel by Simultaneously Inhibiting the Activity of STAT3 and the Interaction of STAT3 with Stathmin. Biochem. Pharmacol..

[115] Yan Y., Lai Y., Tang M., Wang S., Xiao G., Mu Y., Wu X., Zhao A.Z., Li F. (2026). FKBP38 Expression Level Dictates Paclitaxel Resistance via Interacting with Bcl-2 in Endometrial Cancer. Biochem. Pharmacol..

[116] Shi X., Dou Y., Zhou K., Huo J., Yang T., Qin T., Liu W., Wang S., Yang D., Chang L. (2017). Targeting the Bcl-2 Family and P-Glycoprotein Reverses Paclitaxel Resistance in Human Esophageal Carcinoma Cell Line. Biomed. Pharmacother..

[117] Dhadve A., Ray P. (2021). An Active RUNX1-ID1/ID3 Axis Governs Differentiation and Chemoresistance of Cancer Stem Cell Population in Epithelial Ovarian Cancer Cells. Biocell.

[118] Nicolaides N.C., Chrousos G.P. (2023). The Human Glucocorticoid Receptor. Vitam. Horm..

[119] Wang W., Li Z., Zhao J., Chang M., Zhang Y., Shen X., Yue J., Schorge J., Zhang W. (2026). Targeting Glucocorticoid Receptor Signaling in Platinum-Resistant Ovarian Cancer: Translational Rationale and Clinical Advances Following the ROSELLA Trial. Oncol. Rev..

[120] Buonaiuto R., Neola G., Cecere S.C., Caltavituro A., Cefaliello A., Pietroluongo E., De Placido P., Giuliano M., Arpino G., De Angelis C. (2023). Glucocorticoid Receptor and Ovarian Cancer: From Biology to Therapeutic Intervention. Biomolecules.

[121] Mayayo-Peralta I., Zwart W., Prekovic S. (2021). Duality of Glucocorticoid Action in Cancer: Tumor-Suppressor or Oncogene?. Endocr.-Relat. Cancer.

[122] Zhang C., Wenger T., Mattern J., Ilea S., Frey C., Gutwein P., Altevogt P., Bodenmüller W., Gassler N., Schnabel P.A. (2007). Clinical and Mechanistic Aspects of Glucocorticoid-Induced Chemotherapy Resistance in the Majority of Solid Tumors. Cancer Biol. Ther..

[123] Zhang C., Marmé A., Wenger T., Gutwein P., Edler L., Rittgen W., Debatin K.-M., Altevogt P., Mattern J., Herr I. (2006). Glucocorticoid-Mediated Inhibition of Chemotherapy in Ovarian Carcinomas. Int. J. Oncol..

[124] Greenstein A.E., Hunt H.J. (2021). Glucocorticoid Receptor Antagonism Promotes Apoptosis in Solid Tumor Cells. Oncotarget.

[125] Liu B., Deng W.-Z., Hu W.-H., Lu R.-X., Zhang Q.-Y., Gao C.-F., Huang X.-J., Liao W.-G., Gao J., Liu Y. (2025). Psychological Stress-Activated NR3C1/NUPR1 Axis Promotes Ovarian Tumor Metastasis. Acta Pharm. Sin. B.

[126] Rivera-López Y.A., Torres-Rosado A.P., Casiano-Martínez J.A., Meléndez-Rodríguez L.L., Imad-Hamad R., Hernández-Carrasquillo S.M., Rivera-Pérez L.M., Ortiz-León M., Torres-Rodríguez O.I., Aquino-Acevedo A.N. (2025). Chronic Stress Drives Ovarian Cancer Progression via Myeloid-Derived Suppressor Cells Infiltration and Notch Signaling Pathway Activation. Front. Immunol..

[127] Schweer D., McAtee A., Neupane K., Richards C., Ueland F., Kolesar J. (2022). Tumor-Associated Macrophages and Ovarian Cancer: Implications for Therapy. Cancers.

[128] Yin M., Shen J., Yu S., Fei J., Zhu X., Zhao J., Zhai L., Sadhukhan A., Zhou J. (2019). Tumor-Associated Macrophages (TAMs): A Critical Activator in Ovarian Cancer Metastasis. Onco Targets Ther..

[129] Ho C.J., Gorski S.M. (2019). Molecular Mechanisms Underlying Autophagy-Mediated Treatment Resistance in Cancer. Cancers.

[130] Vera-Ramirez L., Vodnala S.K., Nini R., Hunter K.W., Green J.E. (2018). Autophagy Promotes the Survival of Dormant Breast Cancer Cells and Metastatic Tumour Recurrence. Nat. Commun..

[131] Vessoni A.T., Filippi-Chiela E.C., Menck C.F., Lenz G. (2013). Autophagy and Genomic Integrity. Cell Death Differ..

[132] Eliopoulos A.G., Havaki S., Gorgoulis V.G. (2016). DNA Damage Response and Autophagy: A Meaningful Partnership. Front. Genet..

[133] Amaravadi R., Kimmelman A.C., White E. (2016). Recent Insights into the Function of Autophagy in Cancer. Genes. Dev..

[134] Santana-Codina N., Mancias J.D., Kimmelman A.C. (2017). The Role of Autophagy in Cancer. Annu. Rev. Cancer Biol..

[135] Klionsky D.J., Petroni G., Amaravadi R.K., Baehrecke E.H., Ballabio A., Boya P., Bravo-San Pedro J.M., Cadwell K., Cecconi F., Choi A.M.K. (2021). Autophagy in Major Human Diseases. EMBO J..

[136] Cocco S., Leone A., Roca M.S., Lombardi R., Piezzo M., Caputo R., Ciardiello C., Costantini S., Bruzzese F., Sisalli M.J. (2022). Inhibition of Autophagy by Chloroquine Prevents Resistance to PI3K/AKT Inhibitors and Potentiates Their Antitumor Effect in Combination with Paclitaxel in Triple Negative Breast Cancer Models. J. Transl. Med..

[137] Mei D., Chen B., He B., Liu H., Lin Z., Lin J., Zhang X., Sun N., Zhao L., Wang X. (2019). Actively Priming Autophagic Cell Death with Novel Transferrin Receptor-Targeted Nanomedicine for Synergistic Chemotherapy against Breast Cancer. Acta Pharm. Sin. B.

[138] Qin C., Yang G., Yang J., Ren B., Wang H., Chen G., Zhao F., You L., Wang W., Zhao Y. (2020). Metabolism of Pancreatic Cancer: Paving the Way to Better Anticancer Strategies. Mol. Cancer.

[139] Dong P., Wang F., Taheri M., Xiong Y., Ihira K., Kobayashi N., Konno Y., Yue J., Watari H. (2022). Long Non-Coding RNA TMPO-AS1 Promotes GLUT1-Mediated Glycolysis and Paclitaxel Resistance in Endometrial Cancer Cells by Interacting with miR-140 and miR-143. Front. Oncol..

[140] Andreucci E., Biagioni A., Peri S., Versienti G., Cianchi F., Staderini F., Antonuzzo L., Supuran C.T., Olivo E., Pasqualini E. (2023). The CAIX Inhibitor SLC-0111 Exerts Anti-Cancer Activity on Gastric Cancer Cell Lines and Resensitizes Resistant Cells to 5-Fluorouracil, Taxane-Derived, and Platinum-Based Drugs. Cancer Lett..

[141] Pastorekova S., Gillies R.J. (2019). The Role of Carbonic Anhydrase IX in Cancer Development: Links to Hypoxia, Acidosis, and Beyond. Cancer Metastasis Rev..

[142] Aldera A.P., Govender D. (2021). Carbonic Anhydrase IX: A Regulator of pH and Participant in Carcinogenesis. J. Clin. Pathol..

[143] Xiong Z., Li B.-H., Xie J.-J., Li Y.-N., Zhuang R.-L., Yu S.-L., Xie Z.-X., Peng S.-R., Li Z.-A., Li K.-W. (2025). Cancer-Associated Fibroblasts Regulate Mitochondrial Metabolism and Inhibit Chemosensitivity via ANGPTL4-IQGAP1 Axis in Prostate Cancer. J. Adv. Res..

[144] Yu X., Gao X., Mao X., Shi Z., Zhu B., Xie L., Di S., Jin L. (2020). Knockdown of FOXO6 Inhibits Glycolysis and Reduces Cell Resistance to Paclitaxel in HCC Cells via PI3K/Akt Signaling Pathway. OncoTargets Ther..

[145] Wei L., Sun J., Zhang N., Zheng Y., Wang X., Lv L., Liu J., Xu Y., Shen Y., Yang M. (2020). Noncoding RNAs in Gastric Cancer: Implications for Drug Resistance. Mol. Cancer.

[146] Jiang T., Zhu J., Jiang S., Chen Z., Xu P., Gong R., Zhong C., Cheng Y., Sun X., Yi W. (2023). Targeting lncRNA DDIT4-AS1 Sensitizes Triple Negative Breast Cancer to Chemotherapy via Suppressing of Autophagy. Adv. Sci..

[147] Zhang W., Wu Q., Liu Y., Wang X., Ma C., Zhu W. (2022). LncRNA HOTAIR Promotes Chemoresistance by Facilitating Epithelial to Mesenchymal Transition through miR-29b/PTEN/PI3K Signaling in Cervical Cancer. Cells Tissues Organs.

[148] Gu L., Li Q., Liu H., Lu X., Zhu M. (2020). Long Noncoding RNA TUG1 Promotes Autophagy-Associated Paclitaxel Resistance by Sponging miR-29b-3p in Ovarian Cancer Cells. OncoTargets Ther..

[149] Tang L., Chen Y., Chen H., Jiang P., Yan L., Mo D., Tang X., Yan F. (2020). DCST1-AS1 Promotes TGF-β-Induced Epithelial-Mesenchymal Transition and Enhances Chemoresistance in Triple-Negative Breast Cancer Cells via ANXA1. Front. Oncol..

[150] Wang Y., Chen C. (2022). lncRNA-DANCR Promotes Taxol Resistance of Prostate Cancer Cells through Modulating the miR-33b-5p-LDHA Axis. Dis. Markers.

[151] Xu J., Xu Y., Ye G., Qiu J. (2023). LncRNA-SNHG1 Promotes Paclitaxel Resistance of Gastric Cancer Cells through Modulating the miR-216b-5p-Hexokianse 2 Axis. J. Chemother..

[152] Markopoulos G.S., Roupakia E., Tokamani M., Chavdoula E., Hatziapostolou M., Polytarchou C., Marcu K.B., Papavassiliou A.G., Sandaltzopoulos R., Kolettas E. (2017). A Step-by-Step microRNA Guide to Cancer Development and Metastasis. Cell. Oncol..

[153] Shukla G.C., Singh J., Barik S. (2011). MicroRNAs: Processing, Maturation, Target Recognition and Regulatory Functions. Mol. Cell. Pharm..

[154] Jin B., Liu Y., Wang H. (2015). Antagonism of miRNA-21 Sensitizes Human Gastric Cancer Cells to Paclitaxel. Cell Biochem. Biophys..

[155] Wang M., Qiu R., Yu S., Xu X., Li G., Gu R., Tan C., Zhu W., Shen B. (2019). Paclitaxel-Resistant Gastric Cancer MGC-803 Cells Promote Epithelial-to-Mesenchymal Transition and Chemoresistance in Paclitaxel-Sensitive Cells via Exosomal Delivery of miR-155-5p. Int. J. Oncol..

[156] Zang H., Li Y., Zhang X., Huang G. (2020). Circ-RNF111 Contributes to Paclitaxel Resistance in Breast Cancer by Elevating E2F3 Expression via miR-140-5p. Thorac. Cancer.

[157] Li X., Han X., Wei P., Yang J., Sun J. (2020). Knockdown of lncRNA CCAT1 Enhances Sensitivity of Paclitaxel in Prostate Cancer via Regulating miR-24-3p and FSCN1. Cancer Biol. Ther..

[158] Xu Y., Lai Y., Cao L., Li Y., Chen G., Chen L., Weng H., Chen T., Wang L., Ye Y. (2021). Human Umbilical Cord Mesenchymal Stem Cells-Derived Exosomal microRNA-451a Represses Epithelial–Mesenchymal Transition of Hepatocellular Carcinoma Cells by Inhibiting ADAM10. RNA Biol..

[159] Li Y., Zhang L., Dong Z., Xu H., Yan L., Wang W., Yang Q., Chen C. (2021). MicroRNA-155-5p Promotes Tumor Progression and Contributes to Paclitaxel Resistance via TP53INP1 in Human Breast Cancer. Pathol. Res. Pract..

[160] Zheng Y., Li Z., Yang S., Wang Y., Luan Z. (2022). CircEXOC6B Suppresses the Proliferation and Motility and Sensitizes Ovarian Cancer Cells to Paclitaxel through miR-376c-3p/FOXO3 Axis. Cancer Biother. Radiopharm..

[161] Ma S., Kong S., Wang F., Ju S. (2020). CircRNAs: Biogenesis, Functions, and Role in Drug-Resistant Tumours. Mol. Cancer.

[162] Zhang S., Cheng J., Quan C., Wen H., Feng Z., Hu Q., Zhu J., Huang Y., Wu X. (2020). circCELSR1 (Hsa-Circ-0063809) Contributes to Paclitaxel Resistance of Ovarian Cancer Cells by Regulating FOXR2 Expression via miR-1252. Mol. Ther. Nucleic Acids.

[163] Xia B., Zhao Z., Wu Y., Wang Y., Zhao Y., Wang J. (2020). Circular RNA circTNPO3 Regulates Paclitaxel Resistance of Ovarian Cancer Cells by miR-1299/NEK2 Signaling Pathway. Mol. Ther. Nucleic Acids.

[164] Liu G., Zhang Z., Song Q., Guo Y., Bao P., Shui H. (2020). Circ_0006528 Contributes to Paclitaxel Resistance of Breast Cancer Cells by Regulating miR-1299/CDK8 Axis. OncoTargets Ther..

[165] Li P.-P., Li R.-G., Huang Y.-Q., Lu J.-P., Zhang W.-J., Wang Z.-Y. (2021). LncRNA OTUD6B-AS1 Promotes Paclitaxel Resistance in Triple Negative Breast Cancer by Regulation of miR-26a-5p/MTDH Pathway-Mediated Autophagy and Genomic Instability. Aging.

[166] Pedersen C.A., Cao M.D., Fleischer T., Rye M.B., Knappskog S., Eikesdal H.P., Lønning P.E., Tost J., Kristensen V.N., Tessem M.-B. (2022). DNA Methylation Changes in Response to Neoadjuvant Chemotherapy Are Associated with Breast Cancer Survival. Breast Cancer Res..

[167] Shi Y., Gong W., Gong X., Wang P., Zhao X. (2020). Genome-Wide DNA Methylation Analysis of Breast Cancer MCF-7/Taxol Cells with MeDIP-Seq. PLoS ONE.

[168] Xiang F., Zhu Z., Zhang M., Wang J., Chen Z., Li X., Zhang T., Gu Q., Wu R., Kang X. (2021). 3,3’-Diindolylmethane Enhances Paclitaxel Sensitivity by Suppressing DNMT1-Mediated KLF4 Methylation in Breast Cancer. Front. Oncol..

[169] Li H., Wang P., Liu J., Liu W., Wu X., Ding J., Kang J., Li J., Lu J., Pan G. (2020). Hypermethylation of lncRNA MEG3 Impairs Chemosensitivity of Breast Cancer Cells. J. Clin. Lab. Anal..

[170] Bouyahya A., Mechchate H., Oumeslakht L., Zeouk I., Aboulaghras S., Balahbib A., Zengin G., Kamal M.A., Gallo M., Montesano D. (2022). The Role of Epigenetic Modifications in Human Cancers and the Use of Natural Compounds as Epidrugs: Mechanistic Pathways and Pharmacodynamic Actions. Biomolecules.

[171] Li W., Wu H., Sui S., Wang Q., Xu S., Pang D. (2021). Targeting Histone Modifications in Breast Cancer: A Precise Weapon on the Way. Front. Cell Dev. Biol..

[172] Li G.-H., Qu Q., Qi T.-T., Teng X.-Q., Zhu H.-H., Wang J.-J., Lu Q., Qu J. (2021). Super-Enhancers: A New Frontier for Epigenetic Modifiers in Cancer Chemoresistance. J. Exp. Clin. Cancer Res. CR.

[173] Replogle J.M., Zhou W., Amaro A.E., McFarland J.M., Villalobos-Ortiz M., Ryan J., Letai A., Yilmaz O., Sheltzer J., Lippard S.J. (2020). Aneuploidy Increases Resistance to Chemotherapeutics by Antagonizing Cell Division. Proc. Natl. Acad. Sci. USA.

[174] Tamura N., Shaikh N., Muliaditan D., Soliman T.N., McGuinness J.R., Maniati E., Moralli D., Durin M.-A., Green C.M., Balkwill F.R. (2020). Specific Mechanisms of Chromosomal Instability Indicate Therapeutic Sensitivities in High-Grade Serous Ovarian Carcinoma. Cancer Res..

[175] Bhatia S., Khanna K.K., Duijf P.H.G. (2024). Targeting Chromosomal Instability and Aneuploidy in Cancer. Trends Pharmacol. Sci..

[176] Lukow D.A., Sausville E.L., Suri P., Chunduri N.K., Wieland A., Leu J., Smith J.C., Girish V., Kumar A.A., Kendall J. (2021). Chromosomal Instability Accelerates the Evolution of Resistance to Anti-Cancer Therapies. Dev. Cell.

[177] Tucker J.B., Carlsen C.L., Scribano C.M., Pattaswamy S.M., Burkard M.E., Weaver B.A. (2024). CENP-E Inhibition Induces Chromosomal Instability and Synergizes with Diverse Microtubule-Targeting Agents in Breast Cancer. Cancer Res..

[178] Shao X., Zhao X., Wang B., Fan J., Wang J., An H. (2025). Tumor Microenvironment Targeted Nano-Drug Delivery Systems for Multidrug Resistant Tumor Therapy. Theranostics.

[179] Bejarano L., Jordāo M.J.C., Joyce J.A. (2021). Therapeutic Targeting of the Tumor Microenvironment. Cancer Discov..

[180] Jin M.-Z., Jin W.-L. (2020). The Updated Landscape of Tumor Microenvironment and Drug Repurposing. Signal Transduct. Target. Ther..

[181] Liu Y., Hu Y., Xue J., Li J., Yi J., Bu J., Zhang Z., Qiu P., Gu X. (2023). Advances in Immunotherapy for Triple-Negative Breast Cancer. Mol. Cancer.

[182] Tian T., Han J., Huang J., Li S., Pang H. (2021). Hypoxia-Induced Intracellular and Extracellular Heat Shock Protein Gp96 Increases Paclitaxel-Resistance and Facilitates Immune Evasion in Breast Cancer. Front. Oncol..

[183] Han J., Yun J., Quan M., Kang W., Jung J.-G., Heo W., Li S., Lee K.J., Son H.-Y., Kim J.H. (2021). JAK2 Regulates Paclitaxel Resistance in Triple Negative Breast Cancers. J. Mol. Med.-JMM.

[184] Yang Y.-I., Wang Y.-Y., Ahn J.-H., Kim B.-H., Choi J.-H. (2022). CCL2 Overexpression Is Associated with Paclitaxel Resistance in Ovarian Cancer Cells via Autocrine Signaling and Macrophage Recruitment. Biomed. Pharmacother..

[185] Puhr M., Hoefer J., Schäfer G., Erb H.H.H., Oh S.J., Klocker H., Heidegger I., Neuwirt H., Culig Z. (2012). Epithelial-to-Mesenchymal Transition Leads to Docetaxel Resistance in Prostate Cancer and Is Mediated by Reduced Expression of miR-200c and miR-205. Am. J. Pathol..

[186] Khajavirad M.H., Maharati A., Taghehchian N., Taghavinia F., Moghbeli M. (2025). Role of VOPP1 in Regulation of Paclitaxel Response and EMT Process during Ovarian Tumor Progression. Mol. Biol. Res. Commun..

[187] Sofi S., Jan N., Masoodi G., Jan S., Ahmad S.N., Mehraj U., Mir M.A. (2026). Adapalene Inhibits the Activity of CD44, a Cancer Stem Cell Biomarker in Triple Negative Breast Cancer. Mol. Biol. Rep..

[188] Chatterjee E., Dey B., Sharma A., Dharavath A., Naik A., Singh H., Basak S., Pratyusha B., Ashireddygari V.R., Tammineni P. (2026). Autophagy-Lysosomal Dependency Defines a Vulnerable Physiological State in Drug-Tolerant Persister Cells of Triple-Negative Breast Cancer. Apoptosis Int. J. Program. Cell Death.

[189] Monzó M., Rosell R., Sánchez J.J., Lee J.S., O’Brate A., González-Larriba J.L., Alberola V., Lorenzo J.C., Núñez L., Ro J.Y. (1999). Paclitaxel Resistance in Non-Small-Cell Lung Cancer Associated with Beta-Tubulin Gene Mutations. J. Clin. Oncol. Off. J. Am. Soc. Clin. Oncol..

[190] Rouzier R., Rajan R., Wagner P., Hess K.R., Gold D.L., Stec J., Ayers M., Ross J.S., Zhang P., Buchholz T.A. (2005). Microtubule-Associated Protein Tau: A Marker of Paclitaxel Sensitivity in Breast Cancer. Proc. Natl. Acad. Sci. USA.

[191] Hu S., Wang W., Hu Q., Zheng R., Huang Q., Shi H., Ding X., Wang W., Zhu Z. (2026). Utilization of a UPLC-MS/MS Approach to Elucidate the Role of ABCB1-Mediated Paclitaxel Resistance in Non-Small Cell Lung Cancer Cells. Oncol. Res..

[192] Długosz-Pokorska A., Janecki T., Janecka A., Gach-Janczak K. (2024). New Uracil Analog as Inhibitor/Modulator of ABC Transporters or/and NF-κB in Taxol-Resistant MCF-7/Tx Cell Line. J. Cancer Res. Clin. Oncol..

[193] Lin X., Liao Y., Chen X., Long D., Yu T., Shen F. (2016). Regulation of Oncoprotein 18/Stathmin Signaling by ERK Concerns the Resistance to Taxol in Nonsmall Cell Lung Cancer Cells. Cancer Biother. Radiopharm..

[194] Deng X.-Y., Xu H., Peng Y.-X., Guo Y.-Y., He R.-F., Lu G. (2025). Aurora a Degradation by HSP90 Interactome-Mediating PROTACs in A549 and Paclitaxel-Resistant A549 Cells. Eur. J. Med. Chem..

[195] Wise A.R., Maloney S., Hering A., Zabala S., Richmond G.E., VanKlompenberg M.K., Nair M.T., Prosperi J.R. (2024). Bcl-2 up-Regulation Mediates Taxane Resistance Downstream of APC Loss. Int. J. Mol. Sci..

[196] Wu X., Qiu L., Feng H., Zhang H., Yu H., Du Y., Wu H., Zhu S., Ruan Y., Jiang H. (2022). KHDRBS3 Promotes Paclitaxel Resistance and Induces Glycolysis through Modulated MIR17HG/CLDN6 Signaling in Epithelial Ovarian Cancer. Life Sci..

[197] Singh B., Sarli V.N., Lucci A. (2022). Sensitization of Resistant Breast Cancer Cells with a Jumonji Family Histone Demethylase Inhibitor. Cancers.

[198] Lee S.-E., Kim M.-W., Sim Y.-B., Cheon C., Ko S.-G. (2025). Combination of SH003 and Paclitaxel Modulates Tumor Microenvironment and Inhibits Metastasis of Metastatic Melanoma. Med. Oncol..

[199] English D.P., Roque D.M., Santin A.D. (2013). Class III B-Tubulin Overexpression in Gynecologic Tumors: Implications for the Choice of Microtubule Targeted Agents?. Expert Rev. Anticancer Ther..

[200] Ferrandina G., Mariani M., Andreoli M., Shahabi S., Scambia G., Ferlini C. (2012). Novel Drugs Targeting Microtubules: The Role of Epothilones. Curr. Pharm. Des..

[201] Zhang S., Cui T., Duan Y., Zhang H., Wang B., Chen H., Ni J., Shen Y. (2022). Xiao-ai Lv Radix Tetrastigma Extracts Enhance the Chemosensitivity in Triple-Negative Breast Cancer via Inhibiting PI3K/Akt/mTOR-Mediated Autophagy. Clin. Breast Cancer.

[202] Pellegrini E., Multari G., Gallo F.R., Vecchiotti D., Zazzeroni F., Condello M., Meschini S. (2022). A Natural Product, Voacamine, Sensitizes Paclitaxel-Resistant Human Ovarian Cancer Cells. Toxicol. Appl. Pharmacol..

[203] Biswas S., Mahapatra E., Roy M., Mukherjee S. (2020). PEITC by Regulating Aurora Kinase a Reverses Chemoresistance in Breast Cancer Cells. Indian J. Biochem. Biophys..

[204] Roca M.S., Moccia T., Iannelli F., Testa C., Vitagliano C., Minopoli M., Camerlingo R., Riso G.D., Cecio R.D., Bruzzese F. (2022). HDAC Class I Inhibitor Domatinostat Sensitizes Pancreatic Cancer to Chemotherapy by Targeting Cancer Stem Cell Compartment via FOXM1 Modulation. J. Exp. Clin. Cancer Res..

[205] Nakagawa-Saito Y., Mitobe Y., Suzuki S., Togashi K., Sugai A., Kitanaka C., Okada M. (2023). Domatinostat Targets the FOXM1–Survivin Axis to Reduce the Viability of Ovarian Cancer Cells Alone and in Combination with Chemotherapeutic Agents. Int. J. Mol. Sci..

[206] Pascual-Pasto G., Resa-Pares C., Castillo-Ecija H., Aschero R., Baulenas-Farres M., Vila-Ubach M., Burgueño V., Balaguer-Lluna L., Cuadrado-Vilanova M., Olaciregui N.G. (2023). Low Bcl-2 Is a Robust Biomarker of Sensitivity to Nab-Paclitaxel in Ewing Sarcoma. Biochem. Pharmacol..

[207] Kim G., Jang S.-K., Ahn S.H., Kim S., Park C.S., Seong M.-K., Kim H.-A., Bae S., Lee J.H., Kim H. (2024). Proapoptotic Role of CDK1 in Overcoming Paclitaxel Resistance in Ovarian Cancer Cells in Response to Combined Treatment with Paclitaxel and Duloxetine. Cancer Cell Int..

[208] Rezaei N., Kazem Arki M., Miri-Lavasani Z., Solhi R., Khoramipour M., Rashedi H., Asadzadeh Aghdaei H., Hossein-Khannazer N., Mostafavi E., Vosough M. (2023). Co-Delivery of Doxorubicin and Paclitaxel via Noisome Nanocarriers Attenuates Cancerous Phenotypes in Gastric Cancer Cells. Eur. J. Pharm. Biopharm..

[209] Munster P.N., Greenstein A.E., Fleming G.F., Borazanci E., Sharma M.R., Custodio J.M., Tudor I.C., Pashova H.I., Shepherd S.P., Grauer A. (2022). Overcoming Taxane Resistance: Preclinical and Phase 1 Studies of Relacorilant, a Selective Glucocorticoid Receptor Modulator, with Nab-Paclitaxel in Solid Tumors. Clin. Cancer Res..

[210] Olawaiye A.B., Gladieff L., O’Malley D.M., Kim J.-W., Garbaos G., Salutari V., Gilbert L., Mileshkin L., Devaux A., Hopp E. (2025). Relacorilant and Nab-Paclitaxel in Patients with Platinum-Resistant Ovarian Cancer (ROSELLA): An Open-Label, Randomised, Controlled, Phase 3 Trial. Lancet.

[211] Datta S., Choudhury D., Das A., Mukherjee D.D., Dasgupta M., Bandopadhyay S., Chakrabarti G. (2019). Autophagy Inhibition with Chloroquine Reverts Paclitaxel Resistance and Attenuates Metastatic Potential in Human Nonsmall Lung Adenocarcinoma A549 Cells via ROS Mediated Modulation of β-Catenin Pathway. Apoptosis.

[212] Hou Z., Lin S., Du T., Wang M., Wang W., You S., Xue N., Liu Y., Ji M., Xu H. (2023). S-72, a Novel Orally Available Tubulin Inhibitor, Overcomes Paclitaxel Resistance via Inactivation of the STING Pathway in Breast Cancer. Pharmaceuticals.

[213] Raab M., Rak M., Tesch R., Gasimli K., Becker S., Knapp S., Strebhardt K., Sanhaji M. (2021). The Small-Molecule Inhibitor MRIA9 Reveals Novel Insights into the Cell Cycle Roles of SIK2 in Ovarian Cancer Cells. Cancers.

[214] Lin T.-Y., Gu S.-Y., Lin Y.-H., Shih J.-H., Lin J.-H., Chou T.-Y., Lee Y.-C., Chang S.-F., Lang Y.-D. (2024). Paclitaxel-Resistance Facilitates Glycolytic Metabolism via Hexokinase-2-Regulated ABC and SLC Transporter Genes in Ovarian Clear Cell Carcinoma. Biomed. Pharmacother..

[215] Guo Y., Yang L., Guo W., Wei L., Zhou Y. (2021). FV-429 Enhances the Efficacy of Paclitaxel in NSCLC by Reprogramming HIF-1α-Modulated FattyAcid Metabolism. Chem.-Biol. Interact..

[216] Mittendorf E.A., Zhang H., Barrios C.H., Saji S., Jung K.H., Hegg R., Koehler A., Sohn J., Iwata H., Telli M.L. (2020). Neoadjuvant Atezolizumab in Combination with Sequential Nab-Paclitaxel and Anthracycline-Based Chemotherapy versus Placebo and Chemotherapy in Patients with Early-Stage Triple-Negative Breast Cancer (IMpassion031): A Randomised, Double-Blind, Phase 3 Trial. Lancet.

[217] Chang Y., Gao X., Jiang Y., Wang J., Liu L., Yan J., Huang G., Yang H. (2024). Alpha-Hederin Reprograms Multi-miRNAs Activity and Overcome Small Extracellular Vesicles-Mediated Paclitaxel Resistance in NSCLC. Front. Pharmacol..

[218] Zhu Y., Wang A., Zhang S., Kim J., Xia J., Zhang F., Wang D., Wang Q., Wang J. (2023). Paclitaxel-Loaded Ginsenoside Rg3 Liposomes for Drug-Resistant Cancer Therapy by Dual Targeting of the Tumor Microenvironment and Cancer Cells. J. Adv. Res..

[219] Villegas C., González-Chavarría I., Burgos V., Iturra-Beiza H., Ulrich H., Paz C. (2023). Epothilones as Natural Compounds for Novel Anticancer Drugs Development. Int. J. Mol. Sci..

[220] Colombo N., Kutarska E., Dimopoulos M., Bae D.-S., Rzepka-Gorska I., Bidzinski M., Scambia G., Engelholm S.A., Joly F., Weber D. (2012). Randomized, Open-Label, Phase III Study Comparing Patupilone (EPO906) with Pegylated Liposomal Doxorubicin in Platinum-Refractory or -Resistant Patients with Recurrent Epithelial Ovarian, Primary Fallopian Tube, or Primary Peritoneal Cancer. J. Clin. Oncol. Off. J. Am. Soc. Clin. Oncol..

[221] Advani R., Fisher G.A., Lum B.L., Hausdorff J., Halsey J., Litchman M., Sikic B.I. (2001). A Phase I Trial of Doxorubicin, Paclitaxel, and Valspodar (PSC 833), a Modulator of Multidrug Resistance. Clin. Cancer Res..

[222] Lhommé C., Joly F., Walker J.L., Lissoni A.A., Nicoletto M.O., Manikhas G.M., Baekelandt M.M.O., Gordon A.N., Fracasso P.M., Mietlowski W.L. (2008). Phase III Study of Valspodar (PSC 833) Combined with Paclitaxel and Carboplatin Compared with Paclitaxel and Carboplatin Alone in Patients with Stage IV or Suboptimally Debulked Stage III Epithelial Ovarian Cancer or Primary Peritoneal Cancer. J. Clin. Oncol. Off. J. Am. Soc. Clin. Oncol..

[223] Brufsky A., Kim S.B., Zvirbule Ž., Eniu A., Mebis J., Sohn J.H., Wongchenko M., Chohan S., Amin R., Yan Y. (2021). A Phase II Randomized Trial of Cobimetinib plus Chemotherapy, with or without Atezolizumab, as First-Line Treatment for Patients with Locally Advanced or Metastatic Triple-Negative Breast Cancer (COLET): Primary Analysis. Ann. Oncol..

[224] Lorusso D., Gladieff L., O’Malley D.M., Kim J.-W., Garbaos G., Fagotti A., Gilbert L., Mileshkin L., Quesada S., Hopp E. (2026). Overall Survival with Relacorilant and Nab-Paclitaxel in Patients with Platinum-Resistant Ovarian Cancer (ROSELLA): A Phase 3 Randomised Controlled Trial. Lancet.

